# Behaviour change strategies for reducing blood pressure-related disease burden: findings from a global implementation research programme

**DOI:** 10.1186/s13012-015-0331-0

**Published:** 2015-11-09

**Authors:** The GACD Hypertension Research Programme, Writing Group, David Peiris, Simon R. Thompson, Andrea Beratarrechea, María Kathia Cárdenas, Francisco Diez-Canseco, Jane Goudge, Joyce Gyamfi, Jemima Hoine Kamano, Vilma Irazola, Claire Johnson, Andre P. Kengne, Ng Kien Keat, J. Jaime Miranda, Sailesh Mohan, Barbara Mukasa, Eleanor Ng, Robby Nieuwlaat, Olugbenga Ogedegbe, Bruce Ovbiagele, Jacob Plange-Rhule, Devarsetty Praveen, Abdul Salam, Margaret Thorogood, Amanda G. Thrift, Rajesh Vedanthan, Salina P. Waddy, Jacqui Webster, Ruth Webster, Karen Yeates, Khalid Yusoff, Hypertension Research Programme members

**Affiliations:** The George Institute for Global Health, The University of Sydney, Sydney, Australia; UCL Institute for Global Health, London, UK; Institute for Clinical Effectiveness and Heath Policy, Buenos Aires, Argentina; Universidad Peruana Cayetano Heredia, Lima, Peru; University of the Witwatersrand, Johannesburg, South Africa; New York University School of Medicine, New York, USA; Moi University, Eldoret, Kenya; South African Medical Research Council, Cape Town, South Africa; Universiti Teknologi Mara, Selangor, Malaysia; Public Health Foundation of India, New Delhi, India; Mildmay Uganda, Lweza, Uganda; Population Health Research Institute, Hamilton, Canada; McMaster University, Hamilton, Canada; Medical University of South Carolina, Charleston, USA; Kwame Nkrumah University of Science and Technology, Kumasi, Ghana; The George Institute for Global Health, New Delhi, India; University of Warwick, Coventry, UK; School of Clinical Sciences at Monash Health, Monash University, Melbourne, Australia; Icahn School of Medicine at Mount Sinai, New York, USA; National Institutes of Health, Bethesda, USA; Queen’s University School of Medicine, Kingston, Canada

**Keywords:** Implementation science, Hypertension, Behaviour change theory, Collaborative research, Low- and middle-income countries

## Abstract

**Background:**

The Global Alliance for Chronic Diseases comprises the majority of the world’s public research funding agencies. It is focussed on implementation research to tackle the burden of chronic diseases in low- and middle-income countries and amongst vulnerable populations in high-income countries. In its inaugural research call, 15 projects were funded, focussing on lowering blood pressure-related disease burden. In this study, we describe a reflexive mapping exercise to identify the behaviour change strategies undertaken in each of these projects.

**Methods:**

Using the Behaviour Change Wheel framework, each team rated the capability, opportunity and motivation of the various actors who were integral to each project (e.g. community members, non-physician health workers and doctors in projects focussed on service delivery). Teams then mapped the interventions they were implementing and determined the principal policy categories in which those interventions were operating. Guidance was provided on the use of Behaviour Change Wheel to support consistency in responses across teams. Ratings were iteratively discussed and refined at several group meetings.

**Results:**

There was marked variation in the perceived capabilities, opportunities and motivation of the various actors who were being targeted for behaviour change strategies. Despite this variation, there was a high degree of synergy in interventions functions with most teams utilising complex interventions involving education, training, enablement, environmental restructuring and persuasion oriented strategies. Similar policy categories were also targeted across teams particularly in the areas of guidelines, communication/marketing and service provision with few teams focussing on fiscal measures, regulation and legislation.

**Conclusions:**

The large variation in preparedness to change behaviour amongst the principal actors across these projects suggests that the interventions themselves will be variably taken up, despite the similarity in approaches taken. The findings highlight the importance of contextual factors in driving success and failure of research programmes. Forthcoming outcome and process evaluations from each project will build on this exploratory work and provide a greater understanding of factors that might influence scale-up of intervention strategies.

**Electronic supplementary material:**

The online version of this article (doi:10.1186/s13012-015-0331-0) contains supplementary material, which is available to authorized users.

## Background

Elevated blood pressure (BP) is the greatest modifiable risk factor for global burden of disease, responsible for approximately 9.4 million deaths annually and about 7 % of disability-adjusted life years (DALYs) [1]. Importantly, the global burden attributable to elevated BP has increased markedly over the last 20 years, from approximately 137 million DALYs in 1990 to 174 million DALYs in 2010, emphasising the epidemiological transition that has occurred during this time period [1]. In addition, the greatest proportion of this burden now rests with low- and middle-income countries (LMICs), with over 80 % of deaths from elevated BP occurring in these regions [2]. The global cost of elevated BP is estimated to reach nearly US$1 trillion over the next decade [3]. Unless adequately controlled, BP-related disease will continue to be responsible for substantial health and economic burden worldwide.

The Global Alliance for Chronic Diseases (GACD) was founded in 2009 by five of the world’s largest national government health research funding agencies. It has since grown to comprise ten national funding agencies that together contribute 80 % of the world’s publicly funded research [4]. Its overall goal is to tackle the burden of chronic diseases in LMICs and amongst vulnerable populations in high-income countries. It seeks to achieve this by systematically building the implementation research evidence base for sound policymaking through targeted research calls that are coordinated across all participating funding agencies.

For its first joint funding initiative, the GACD focussed on the prevention, management and control of elevated BP. The central aims of the research programme are to (1) develop a better understanding of critical barriers and facilitators at local and national levels that affect BP control and to consider how implementation challenges can be overcome, (2) to understand how innovations for BP control can be introduced and scaled-up across a range of settings and (3) to identify what health system elements must be strengthened to improve BP control whilst reducing disparities across population sub-groups. The programme includes 15 projects spanning 15 LMICs and Aboriginal communities in Canada, with approximately US$23 million committed over 5 years. Protocols for several of the funded projects have been published [5–22], and project synopses are available on the GACD website (www.gacd.org) [23].

In this paper, we outline a mapping exercise that was undertaken collaboratively by the 15 research teams. Specific aims are to (1) identify in each project the target behaviour change and the principal actors who are central to achieving change, (2) to use a behaviour change model to determine each team’s perception of the influences on these actors to change behaviour and (3) to map the intervention functions used by each team to promote behaviour change amongst these actors and to determine the principal policy categories in which those interventions are operating. This mapping work was undertaken in the early stages of the 5-year GACD Hypertension Research Programme and serves as a benchmark upon which to gauge progress following the implementation of the component studies.

## Methods

### Participants

A GACD research programme committee comprising at least one high-income country and one LMIC representative from each of the 15 teams was formed in 2012. At the committee’s first meeting in late 2012, research team representatives commenced collaborative work to better understand the local contexts in which we were working and to determine the degree of alignment of our various studies and interventions. Although there is a high degree of variation in the settings, there were many similarities in approach. All funded teams are seeking to change prevailing behaviours at the level of individuals (e.g. community members, health service attendees and health care providers), organisations (health care services, educational institutions, food providers) and systems (local and large scale policy initiatives). A working group comprising representatives from most of the 15 research teams and the GACD funding agencies was consequently formed to undertake a mapping exercise to better understand and synthesise the behaviour change dynamics in these projects.

### Behaviour change framework

**Fig. 1:**
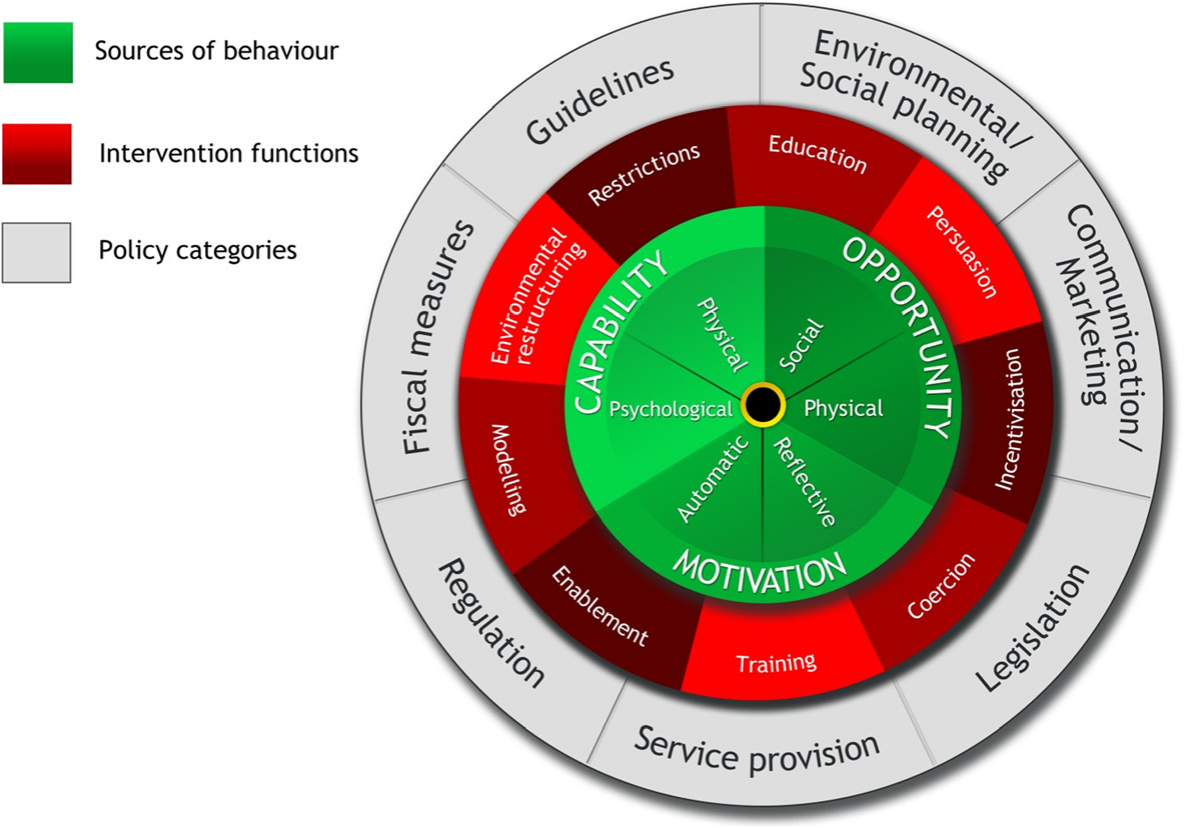
The Behaviour Change Wheel [24]. Notes: At the centre of the wheel are the COM-B model components. Capability refers to an individual’s physical and psychological capacity (e.g. comprehension, literacy, reasoning) to engage in the activity concerned. It includes having the necessary knowledge and skills to enact the target behaviour. Motivation refers to brain processes that energise and direct behaviour. Includes automatic processes characterised by habit, emotions and impulses as well as reflective processes involving analytical decision making, making plans and evaluating them. Opportunity refers to the factors that lie outside the individual that make behaviour change possible or prompt it. Can be physical opportunities afforded by the environment in which people live or social opportunity which is affected by the cultural milieu in which we think about things, words we use and concepts that make up our language [24]

The working group drew on Michie and colleagues’ Behaviour Change Wheel framework to systematically map the respective behaviour change “targets” of each project (Fig. 1) [24]. This framework comprises a behaviour system at the hub involving three essential conditions: capability, opportunity and motivation (the COM-B model). Encircling this hub are nine intervention functions, aimed at addressing deficits in one or more of these conditions. A larger wheel surrounds these intervention functions and comprises seven policy categories. These policy categories are broader population-level strategies that could enable those interventions to occur. The intervention and policy codes within this framework are provided in Table 1.

**Table 1:** Intervention functions and policy categories in the Behaviour Change Wheel

Interventions	
Education	Increasing knowledge or understanding
Persuasion	Using communication to induce positive or negative feelings or stimulate action
Incentivisation	Creating expectation of reward
Enablement	Increasing means/reducing barriers to increase capability or opportunity beyond education, training and environmental restructuring respectively
Training	Imparting skills
Coercion	Creating expectation of punishment or cost
Restriction	Using rules to reduce the opportunity to engage in the target behaviour (or increase the opportunity to engage in competing behaviours)
Environmental restructuring	Changing the physical and social context
Modelling	Providing an example for people to aspire to or imitate
Policies	
Communication/marketing	Using print, electronic, telephonic or broadcast media
Guidelines	Creating documents that recommend or mandate practice
Fiscal measures	Using the tax system to reduce or increase the financial cost
Regulation	Establishing rules or principles of behaviour or practice
Legislation	Making or changing laws
Environmental/social planning	Designing and/or controlling the physical or social environment
Service provision	Delivering a service

### Survey procedure

A survey tool was developed in which we asked research teams to conduct the following activities. First, teams outlined in one sentence what they considered to be the principal behaviour change target for their project. Second, they used the Behaviour Change Wheel to identify the current capabilities, opportunities and motivations of the principal “actors” that must be engaged in order to achieve those targets. A simple, 3-point rating was provided to gauge the strengths of each actor within each COM-B component (e.g. community health worker capability to improve BP control in the clinic could be rated as low, medium or high). Although there are sub-components to the COM-B model (e.g. physical and social opportunity), we did not provide ratings at this level in order to minimise complexity for many research teams who were new to this model. Third, each team mapped their proposed interventions against each of the Behaviour Change Wheel intervention functions. Teams were encouraged to classify all of their intervention strategies that they were deploying regardless of whether they were directly related to the target behaviour change. Fourth, each team identified the principal policy categories in which those interventions were either actively influencing or being influenced. Researchers were provided with the definitions for each of the intervention and policy categories as part of the survey tool (Table 1). These definitions and guidance on how to categorise interventions followed the principles in the paper by Michie and colleagues [24].

To assist teams in completing the survey tool, several strategies were undertaken. A working group, comprising several researchers and representatives from the GACD funding agencies, was convened to guide development of the survey tool. One research team who had previously used the Behaviour Change Wheel theory completed the survey tool for their project and provided this as an exemplar for the other teams. The principal investigator from this team also conducted teleconferences with each of the research teams to guide them through use of the tool and to ensure there was a consistent understanding of the survey terms. Teams were encouraged to complete the tool as a group activity, to closely engage local stakeholders and to draw on existing research conducted in the regions they were working. Because such activities are inherently reductive in nature, we also encouraged teams to provide a more detailed narrative description of key contextual factors that influenced their ratings. Table 2 outlines how the process was conducted in one of the projects.

**Table 2:** SMARThealth India- Project 7 (IND 7)

**Project overview**	
SMARThealth India uses mobile technologies to provide village-based, non-physician health workers with personalised clinical decision support to guide cardiovascular disease (CVD) risk assessment and management. The system is being tested in rural villages in Andhra Pradesh, India. It is integrated with government primary health care centres. Individuals identified at high CVD risk are referred to the treating doctor for ongoing management and follow-up. The doctor also has access to the decision support tools and patients are provided with interactive voice prompts to support ongoing care and follow-up. The system is being tested in a stepped-wedge cluster randomised controlled trial involving 18 primary health care centres, 54 villages and around 15,000 individuals at high CVD risk. The primary outcome is improvements in the proportion of people at high CVD risk who are achieving national guidelines blood pressure targets.	
**Previous research conducted to inform this work**	
The research team has been working in this region for the past decade. Previous studies had been conducted documenting the rise in blood pressure related disease burden in the region and gaps in access to recommended treatments had been quantified [34]. An intervention trial had also been conducted which found that non-physician health workers could perform routine CVD risk assessments to the level of a physician using a simple paper-based algorithm chart [35].	
**Pilot study**	
Building on this work, a prototype tablet based decision support 'app' was developed and trialed for use by 11 non-physician health workers and three government doctors for around 200 patients. The COM-B model was used to guide the evaluation [22]. The qualitative component identified three inter-related interview themes: (1) the decision support technology had potential to change prevailing health care models, (2) shifting tasks traditionally performed by a doctor to the community health worker was the central driver of change, and (3) despite high acceptability by end users, actual healthcare transformation was substantially limited by system-level barriers such as patient access to doctors and medicines.	
**Completion of the survey tool**	
On the basis of the above information the SMARThealth research team met via teleconference to complete the survey. The target behaviour change is to improve blood pressure control amongst people at high CVD risk. A consensus approach was taken to determine the ratings and this was informed by the recently completed pilot evaluation. Doctor capability was rated high, however, motivation and opportunity were rated low. The pilot evaluation found that working conditions, salary and competing priorities were all factors that limited doctors from improving blood pressure control in the target population. For health workers capability was low as most health workers had no previous experience in conducting CVD risk assessments, however, motivation was assessed as high as previous research demonstrated high levels of interest in expanding current roles to include chronic disease screening and prevention. Current opportunities, however, to do this are low as there are few chronic disease training programs for this workforce. Community capability and opportunity were rated low as previous studies have demonstrated large health literacy gaps and major shortfalls in people's ability to access health care. Motivation to engage in the primary health care sector was rated medium as community members interviewed during the pilot had varying confidence in the ability of this sector to meet their healthcare needs.	

### Analysis

Upon completion of the first survey draft, the preliminary results were presented at the second annual steering committee meeting in late 2013 which all research teams attended. This provided an opportunity for further engagement in refining the responses made in the tool. Further, because projects were at different stages when the initial mapping work was done, several teams adapted their responses on the basis of exploratory research they had conducted in the early stages of their projects. In this way each team’s survey response was iteratively revised over a 12-month period. The findings were again presented at the 2014 annual steering committee meeting. General consensus on the key messages was obtained, and the working group drafted the findings for publication.

## Results

The 15 research projects, their proposed behaviour change targets and a brief summary of the interventions proposed are outlined in Table 3. Two teams (Tanzania/Canada and South Africa/Uganda) completed separate templates for each region they were working in due to a high degree of variation in the principal actors operating in each region of the same project. Projects broadly aligned into two topic areas—health care delivery interventions (the majority) and salt reduction/substitute interventions. For the healthcare delivery interventions, a range of multifaceted strategies are being tested to improve screening, detection, management and follow-up of individuals with elevated BP. These include task-shifting of doctor responsibilities to non-physician health workers (NPHWs) (nurses, community health workers and peer lay workers) and integrating primary health care clinics with existing models of care for other diseases (e.g. HIV clinics). Several project teams are also incorporating an “mHealth” component which comprises multi-dimensional elements including provider, patient and administrative applications that are accessible via a mobile device for the provision and receipt of healthcare. The remaining projects are targeting salt reduction at both organisational levels (schools and communities) and at policy levels. Different strategies being implemented include salt substitutes, peer-led school education programmes, policy interventions around salt levels in foods and meals and community campaigns.

**Table 3:** Funded research projects, behaviour change targets and planned interventions. Restrictions; Education; Persuasion; Incentivisation; Coercion; Training; Enablement; Modelling; Environmental restructuring. Guidelines; Environment/social planning; Communication/marketing; Legislation; Service provision; Regulation; Fiscal measures. “Partially” encompasses categories where some elements of a particular intervention characteristic are present, but it was not the dominant feature

Project code (team no)	Funding agency	Target countries/regions	Target behaviour change	Intervention	Intervention function	Policy categories
Interventions involving mobile health technologies						
IND 7	NHMRC	India-Andhra Pradesh	Improved BP control amongst people at high cardiovascular disease risk	A primary health care mHealth system for use by NPHWs and government primary health care centre doctors	• Education	• Guidelines
					• Persuasion	• Communication/ marketing (partially)
					• Incentivisation	• Service provision (partially)
					• Training	
					• Enablement	
					• Environmental restructuring	
TZA 3/CAN 3	CIHR, GCC, IDRC, and CSN	Tanzania/Canada	Improved HT control through improved screening, lifestyle changes and medication use	Primary health care intervention involving linkage of primary health care workers and patients via a short message system (SMS) mHealth system, combined with contextually and culturally specific training programmes for health care workers in the two settings	• Education	• Guidelines
					• Training	• Environment/social planning
					• Persuasion (partially)	• Communication/marketing
					• Incentivisation (partially)	• Legislation (partially)
					• Enablement (partially)	• Service provision (partially)
					• Environmental restructuring (partially)	• Regulation (partially)
COL/MYS 2	CIHR, GCC, IDRC, and CSN	Colombia/Malaysia	Improved BP control through improved screening, lifestyle changes and medication use	Primary health care programme for cardiovascular disease risk assessment, treatment and control involving: (1) simplified algorithms implemented by NPHWs and supported by e-health technologies; (2) initiation of evidence based medications; (3) treatment supports to optimise long-term medication and lifestyle adherence; and (4) macro-policy initiatives to support sustainability	• Education	• Guidelines
					• Persuasion	• Environment/social planning
					• Training	
					• Enablement	• Communication/marketing
						• Service provision
						• Legislation (partially)
KEN 13	NIH (NHLBI)	Kenya	Linking and retaining hypertensive individuals to hypertensive care	A behavioural communication strategy and use of mHealth tools to improve linkage into health care and optimal BP control	• Education	• Guidelines
					• Persuasion	• Communication/marketing
					• Training	• Service provision
					• Enablement	
					• Environmental restructuring	
					• Incentivisation (partially)	
ARG 14	NIH (NHLBI)	Argentina	Improved BP control amongst hypertensive subjects via improved medication adherence, home monitoring and a lifestyle modification programme	Primary health care intervention comprising provider education, a home-based lifestyle and BP monitoring consultation for patients and their families delivered by NPHWs and a mHealth intervention	• Education	• Guidelines
					• Persuasion	• Service provision
					• Incentivisation	
					• Training	
					• Enablement	
					• Environmental restructuring	
Innovative health care delivery strategies						
NGA 15	NIH (NINDS)	Nigeria	Empowering patients who have had a stroke to improve their adherence to medicines and recommended health care visits	A new model of care comprising a stroke patient report card, SMS messages from care providers, and in-clinic educational video sessions	• Education	• Guidelines
					• Persuasion	• Communication/marketing
ZAF 1	CIHR, GCC, IDRC, and CSN	South Africa—Western Cape/Uganda	Improved control of BP amongst people with HIV	N/A (Observational study)	N/A	N/A
UGA 1						
ZAF 5	MRC	South Africa-Mpumalanga	Changing clinic systems and behaviour of health professionals in the clinic	Primary health care intervention in which a clinic based lay health worker will support outreach teams to improve access and quality of care for patients with elevated BP	• Education	• Service provision
					• Training	
					• Enablement	
					• Environmental restructuring	
					• Persuasion (partially)	
IND 6	NHMRC	India-3 rural regions	Improved control of BP amongst rural-dwelling people with hypertension	Peer group based support incorporating monitoring and education, and a non-physician health care facilitator. Health system and workforce strengthening.	• Education	• Guidelines
					• Persuasion	• Environment/social planning
					• Incentivisation	• Communication/marketing
					• Training	• Service provision
					• Enablement	• Guidelines (partially)
					• Modelling	
					• Environmental	
					• restructuring	
IND 8	NHMRC	India/Sri Lanka-fixed dose combination BP-lowering pill	Improve prescriber and patient uptake of BP-lowering medication	Outpatient clinic trial of a low dose 3-in-1 low cost BP-lowering pill compared with usual treatment regimes. 700 patients will be randomised to treatment with a triple low dose BP-lowering medication or routine management of hypertension according to usual practice. Primary outcome is proportion reaching target at 6 months.	• Enablement	
					• Environmental restructuring	
GHA 12	NIH (NHLBI)	Ghana	Improved BP control amongst patients with uncontrolled hypertension who receive care in community-based primary care practices	Comparative effectiveness study of an World Health Organisation package of interventions involving task-shifting to Community Health Nurses versus provision of health insurance coverage	• Education	• Guidelines
					• Persuasion	• Environment/social planning
					• Incentivisation	• Communication/marketing
					• Training	• Service provision
					• Enablement	• Regulation
					• Modelling	
					• Environmental restructuring	
Salt reduction strategies						
IND 9	NHMRC	India—national	Reduction in dietary intake of salt and reduction of salt levels in foods and meals through the development of local and national policies for salt reduction	N/A (Observational study)	N/A	• Guidelines
						• Communication/marketing
FJI/ WSM 10	NHMRC	Pacific Islands	Reduce salt use	Multi-pronged cross sectoral programmes targeting community-wide salt reduction	• Education	• Guidelines
					• Training	• Communication/marketing
					• Environmental restructuring	• Regulation
CHN 4	MRC	China	Lowered salt intake in children and their families	A school-based education programme to reduce salt intake in children and their families	• Restrictions	• Guidelines
					• Education	• Environment/social planning
					• Persuasion	• Communication /marketing
					• Incentivisation	• Service provision (partially)
					• Training	
					• Enablement	
					• Modelling	
					• Environmental restructuring	
PER 11	NIH (NHLBI)	Peru	Replacing common salt for a potassium-enriched salt (substitute); incorporating and consuming the salt substitute in the usual diet	A community-wide salt substitution programme involving community, community kitchens, and food suppliers	• Education	• Communication/marketing
					• Persuasion	
					• Incentivisation	
					• Training	
					• Enablement	
					• Modelling	
					• Environmental restructuring	
					• Restrictions (partially)	

**Fig. 2:**
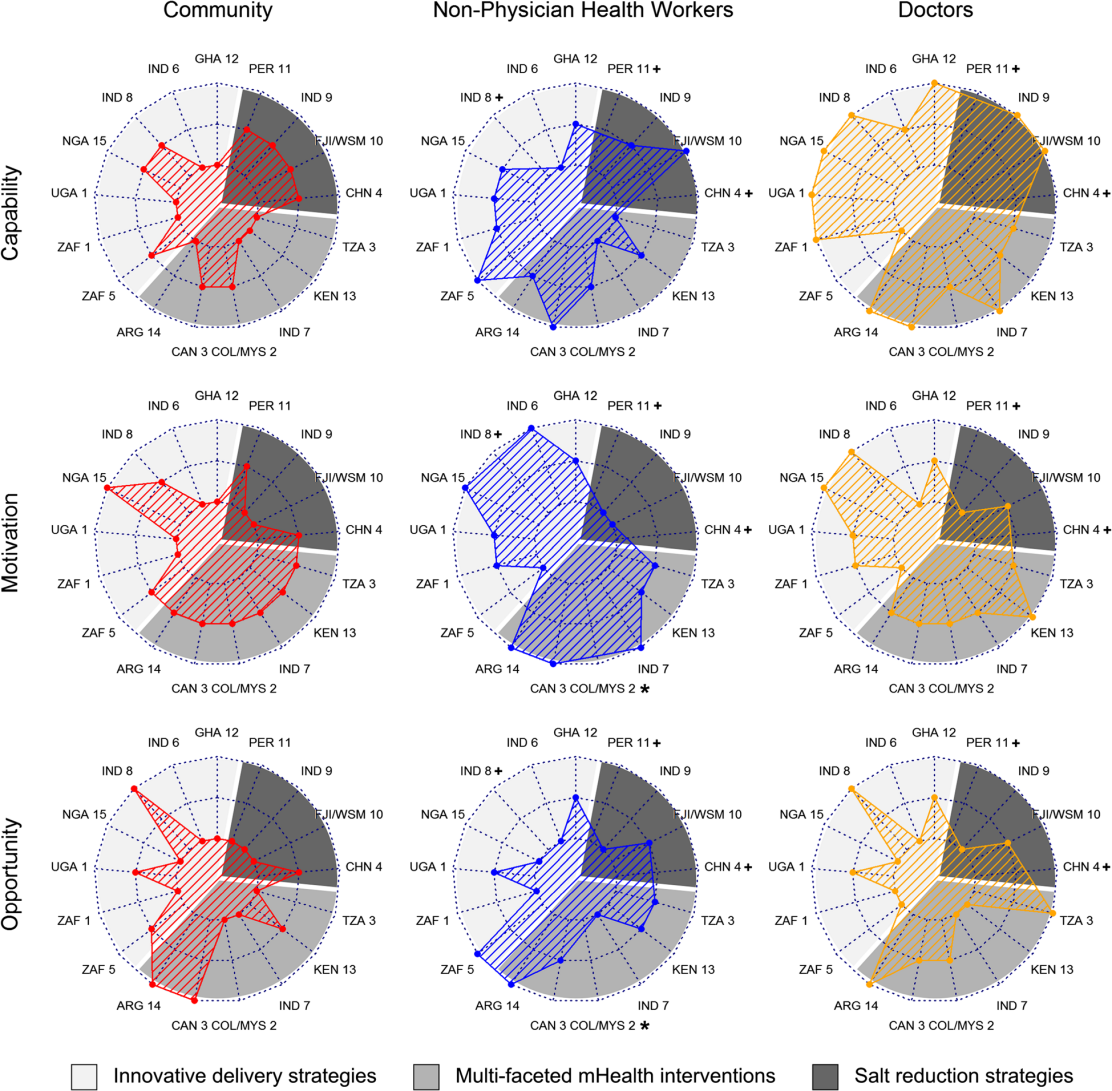
Capability, opportunity and motivation ratings of community members, non-physician health workers and doctors in 15 research projects. + not working with this particular target group. * rating not provided. Notes: (1) Research teams are ordered around the plot by their strategy according to Table 2 and then alphabetically. (2) Each team provided ratings on a 3-point scale (low (*inner ring*), medium (*middle ring*) and high (*outer ring*)) for each of the “actors” with whom they were targeting their interventions. This was done for each of the COM-B components (capability, opportunity and motivation). The more peripheral the location, the higher the rating. (3) Teams provided additional explanatory text providing contextual information for how they arrived at their ratings. Please see the supplemental online files. (4) Several projects are targeting other “actors”, and these are not included in these figures (e.g. for the India triple pill project, ratings are left blank for NPHWs as this project is not engaging with these groups; for the Peru salt project, ratings are left blank for doctors and NPHWs as this project is mainly engaging with the community, community kitchens and retail food outlets.) Please refer to the individual project templates in the supplemental online files to view their ratings for these other actors

Figure 2 outlines the results of the behaviour change mapping exercise using radial charts to preserve specificity for each team’s ratings. A smaller “web” indicates low ratings for the COM-B components across all projects whilst a larger web indicates higher ratings. For most projects, there were three principal actors (community, NPHWs and doctors), and we focussed on these groups in the results. In some projects, other actors were also identified, particularly for the salt reduction projects where community organisations, food outlets and schools were the main focus of activity rather than health services. Ratings for these groups and the changes made by groups over time to their ratings are outlined in Additional file 1: Figure S1. A detailed summary of each team’s contextual information for their survey responses is also available in Additional files 2, 3, 4, 5, 6, 7, 8, 9, 10, 11, 12, 13, 14, 15, 16, 17 and 18.

Most of the radial charts in Fig. 2 are highly irregular in shape, indicating wide variability in ratings across projects and no clear overall pattern. The one exception to this was that almost all projects rated doctor capability to engage in the target behaviour as medium to high, reflecting higher levels of training, knowledge and skills in the topic area. NPHW capability was variable with eight projects assigning a medium rating, three projects a high rating and three projects a low rating. All teams rated community capability as low to medium. There was a high degree of variability in motivation ratings across all regions, interventions and actors, although generally, community motivation tended to be rated low to medium. Opportunity for behaviour change tended to be rated low to medium for the majority of projects for all actors with one noteworthy exception in Argentina in which opportunity was rated high for all actors. Apart from the mhealth projects having similar motivation ratings across actors, there were few other clear patterns of COM-B ratings by intervention type (salt, mHealth and innovative delivery strategies) (Fig. 2).

**Fig. 3:**
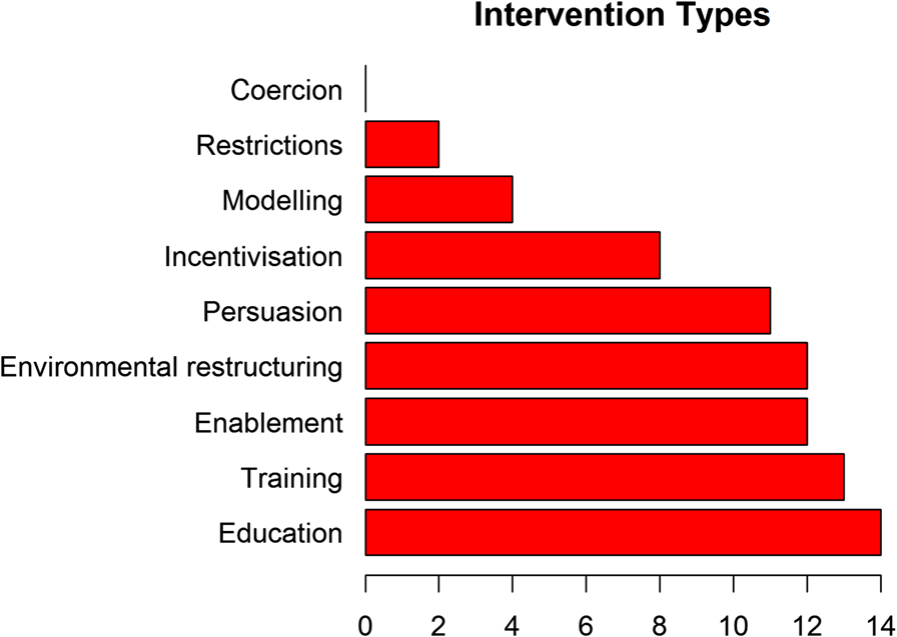
Intervention functions for the 15 research projects using the Behaviour Change Wheel framework

**Fig. 4:**
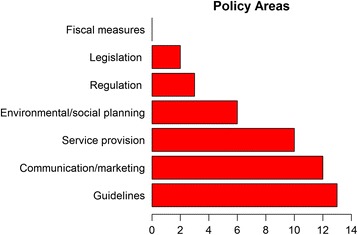
Policy categories for the 15 research projects using the Behaviour Change Wheel framework

Figure 3 outlines the number of projects that are targeting particular intervention functions. All projects are engaging in multiple intervention functions (median = 6, range 2–8) with education, training, enablement, environmental restructuring and persuasion being the most common strategies deployed. These were particular strong elements in the projects involving task-sharing with non-physician, frontline health workers. Several projects are focussing on environmental restructuring either through different health care delivery models or through changing access to salt in the food supply. Fewer projects are using incentivisation, restrictions and modelling. No projects are using coercion strategies. Figure 4 shows the policy categories in which the proposed interventions are interacting. Again most projects are operating across multiple policy categories (median number of categories = 3, range 1–6) with guidelines, service provision and communication/marketing being the most common. Few projects are engaged in more structural policy categories such as legislation, regulation and social planning. No projects are employing fiscal measures.

## Discussion

This exploratory study of a global implementation research programme used behaviour change theory to map activities for 15 research projects operating in 15 countries. In doing so, we were able to synthesise the approaches being taken to tackle BP-related disease in a wide range of settings. The principal finding from the mapping activity was the marked variation in the perceived capabilities, opportunities and motivations of the various actors we aim to influence through these projects. This suggests that the ability to change behaviour differs greatly across regions and between actors. Although there was a great variation in behaviour change ratings, there was a high degree of synergy in the types of projects that have been funded through this programme. Most projects are multifaceted, focusing on education, enablement and training strategies to promote behaviour change and most projects are drawing on “softer” policy levers such as guidelines, communication/marketing and service provision rather than restrictive policies such as regulation, fiscal measures and legislation. This possibly reflects the nature of the initial GACD funding request for proposals, the funding amounts available and the duration of the grants.

The main strength of this study is the collaborative nature of the work involving all 15 teams funded by the GACD. By drawing on the collective experiences of researchers working in diverse regions, we were able to capitalise on research that had been conducted prior to these specific GACD-funded projects. Although there are examples of theme-specific bilateral funding agency research programmes [25–27], the GACD is a multilateral funding initiative focussed on implementation science principles. There are few examples of such global implementation programmes [28], and therefore, this programme provides an opportunity to synthesise common elements across research projects. This behaviour change mapping activity facilitated early cross-fertilisation of ideas between teams. New working groups focussed on other topic areas are now developing, and it is expected the group discussions will further promote the development of peer-to-peer networks and synthesis of activities that are common to multiple groups and regions. Another feature of the GACD is close engagement between researchers and the funding agencies. The activities of the Hypertension Research Programme are already being used to inform future research programme funding calls.

Although the iterative and self-reflexive nature of the work was generally considered to be a positive experience by the participating researchers, it also raises the possibility of potential limitations. First, the use of a uniform mapping template required study teams to work within the constraints of the Behaviour Change Wheel framework. Given most teams did not use the framework in the original designing of their interventions, in effect we were “retro-fitting” it to provide an overall classification structure for the 15 projects. There is the potential that such an exercise resulted in teams forcing “square pegs into round holes” with a resultant lack of attention to individual project specifics. Although the framework fitted particularly well for health care delivery interventions, it was less easy to apply in the more policy-oriented interventions (such as salt reduction) and non-intervention-focussed observational studies. Second, although there were specific efforts undertaken to ensure consistent understanding of the Behaviour Change Wheel framework, it is possible that teams interpreted definitions of the categories differently. Further, it must be emphasised that we based the study on researcher perceptions which introduces a somewhat subjective element to the exercise. This is mitigated, in part, by the fact that the research teams have conducted substantial prior empirical work to help inform these ratings (e.g. see Table 2). Third, the reductive nature of the exercise prevented us from providing a more expansive justification for particular ratings for the COM-B model components although teams did provide additional data on the contextual factors that influenced particular choices. For example, it is possible that rating individual actors was too simplistic, neglecting the importance of interaction between actors (e.g. teamwork in the health centre) and the dynamic interplay between various actor groups in influencing the likelihood of an intervention being implemented.

Although we expected similar actors to exhibit similar COM-B ratings across projects, the high degree of variability we observed suggests that the actors themselves and the local contexts in which they operate may be different. Further, the interventions themselves, although similar in nature are complex, and the extent to which they are tailored to the regions in which they are working are likely to be a critical driver of outcomes. We postulate that these different actor-context-intervention constellations will be key drivers of why similar interventions may produce different outcomes and why similar actors may differentially adopt those interventions. When taken as a whole, the GACD research programme, therefore, has potential to contribute important insights into the factors that influence particular implementation strategies.

The different approaches highlight the importance of detailed process evaluations to better understand the reach, fidelity of adoption and maintenance of interventions over time rather than simply focussing on whether the interventions “worked” or not. The UK Medical Research Council has recently published a process evaluation framework for complex interventions which companions its 2008 complex interventions guidance [29, 30]. It draws substantially from realist evaluation, a “mid-range” theory, which is increasingly being used to understand the interaction between context, mechanism and outcome for particular programmes [31]. Rather than viewing interventions as “magic bullets that will hit their target”, realist evaluation seeks to answer the question “what works, for whom, and in what circumstances?” [32, 33]. The GACD research programme provides opportunities for research teams to address these questions collectively. A crucial initial step in conducting a realist evaluation is to make explicit the underlying assumptions about how an intervention is meant to work and what impact it is expected to have. The next stage is to review, with the empirical data arising from the projects, whether these theories are supported, contradicted or need modification in some way. This process will enable us to expose the generative mechanisms of the intervention and its interaction with particular contextual features (e.g. roles and relationships of personnel at health services, service infrastructure, who pays for healthcare, access to essential medicines etc.). Several GACD teams have integrated process evaluations as part of their overall project aims, and some are explicitly using realist evaluation frameworks.

## Conclusions

In this paper, we have collectively used a theoretical framework, the Behaviour Change Wheel, to make explicit our hypothesised intervention effects. Subsequent work will be conducted toward the end of the programme when we will re-visit the mapping exercise undertaken here and use the empirical data from each project to derive a more nuanced analysis of what actually happened. There are important practical policy implications arising from these activities. By making explicit the types of context-mechanism-outcome configurations that are associated with success and failure, policy makers will be better informed on what and how to scale-up in non-research settings. Such activities may also yield important insights into potential policy changes that might need to be enacted for such interventions to be successful at scale (e.g. role expansion for NPHWs). Finally, the outcomes of such activities are instructive for funding agencies and will help inform how global research programmes can be developed into “implementation laboratories”, thereby, delivering new knowledge that extends beyond the individual research projects that they fund.

### Availability of supporting data

The behaviour change surveys completed by each team are available as supplementary materials.

## Additional files

Additional file 1: Figure S1. Capability, opportunity, and motivation ratings of community members, non-physician health workers and doctors in 15 research projects at initial and subsequent assessment. Additional file 2:
**UGA 1 Assessment.** Utilizing HIV/AIDS Infrastructure as a Gateway to Chronic Care of Hypertension in Africa - Uganda (UGA 1). Additional file 3:
**ZAF 1 Assessment.** Utilizing HIV/AIDS Infrastructure as a Gateway to Chronic Care of Hypertension in Africa - South Africa (ZAF1). Additional file 4:
**COL/MYS 2 Assessment.** Developing an Innovative Strategy for Hypertension Detection, Treatment and Control in Two MiddleIncome Countries (Hypertension Outcomes Prevention and Evaluation: HOPE -4) (COL/MYS 2). Additional file 5:
**CAN 3 Assessment.** DREAM-GLOBAL: Diagnosing hypeRtension - Engaging Action and Management in Getting LOwer Bp in Aboriginal and LMIC - Canada (CAN 3). Additional file 6:
**TZA 3 Assessment.** DREAM-GLOBAL: Diagnosing hypeRtension - Engaging Action and Management in Getting LOwer Bp in Aboriginal and LMIC - Tanzania (TZA 3). Additional file 7:
**CHN 4 Assessment.** A School-Based Education Programme to Reduce Salt Intake in Children and their Families (CHN 4). Additional file 8:
**ZAF 5 Assessment.** Treating Hypertension in Rural South Africa: Strengthening Community- Based Outreach Services for Integrated Chronic Care (ZAF 5). Additional file 9:
**IND 6 Assessment.** Improving the Control of Hypertension in Rural India: Overcoming the Barriers to Diagnosis and Effective Treatment (IND 6). Additional file 10:
**IND 7 Assessment.** A Smartphone-Based Clinical Decision Support System for Primary Health care Workers in Rural India (IND7). Additional file 11:
**IND 8 Assessment.** Randomised Controlled Trial of Early Use of a Simplified Treatment Regimen Incorporating a Half-Dose, Three-in-One Blood Pressure Lowering Pill vs. Usual Care for Improving Hypertension Control in India (IND8). Additional file 12:
**IND 9 Assessment.** Developing the Evidence Base for a National Salt Reduction Programme for India (IND 9). Additional file 13:
**FJI/WSM 10 Assessment.** Cost Effectiveness of Salt Reduction Interventions in Pacific Islands (FJI/WSM 10). Additional file 14:
**PER 11 Assessment.** Launching a Salt Substitute to Reduce Blood Pressure at the Population Level (PER 11). Additional file 15:
**GHA 12 Assessment.** Task Shifting and Blood Pressure Control in Ghana: A Cluster- Randomized Trial (GHA 12). Additional file 16:
**KEN 13 Assessment.** Optimizing Linkage and Retention to Hypertension Care in Rural Kenya (KEN 13). Additional file 17:
**ARG 14 Assessment.** Comprehensive Approach for Hypertension Prevention and Control in Argentina (ARG 14). Additional file 18:
**NGA 15 Assessment.** Tailored Hospital-based Risk Reduction to Impede Vascular Events after Stroke (THRIVES) (NGA 15).

## Abbreviations

GACD: Global Alliance for Chronic Diseases
BP: blood pressure
CIHR: Canadian Institutes of Health Research
CSN: Canadian Stroke Network
DALYs: disability-adjusted life years
GCC: Grand Challenges Canada
IDRC: International Development Research Centre
LMIC: low- and middle-income country
MRC: Medical Research Council—United Kingdom
NHLBI: National Heart, Lung and Blood Institute
NHMRC: National Health and Medical Research Council—Australia
NIH: National Institutes of Health—USA
NINDS: National Institute of Neurological Disorders and Stroke
NPHWs: non-physician health workers
SMS: short message service
