# Supplementary figures and images for: Behaviour change strategies for reducing blood pressure-related disease burden: findings from a global implementation research programme
Source: Implement Sci. 2015 Nov 9;10:158. doi: 10.1186/s13012-015-0331-0 (PMC4638103; doi:10.1186/s13012-015-0331-0)

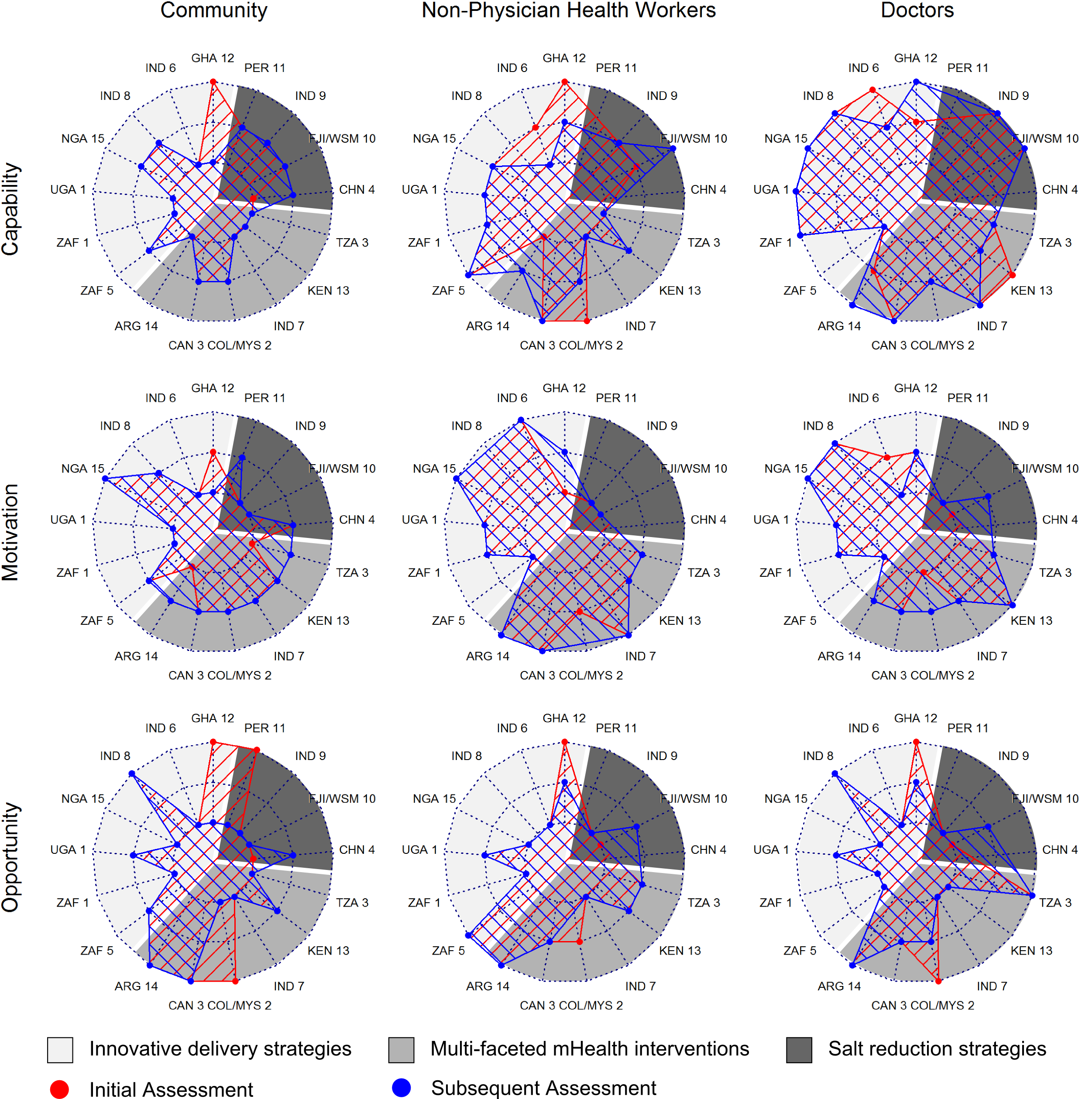

Supplement: Additional file 1: Figure S1. — Capability, opportunity, and motivation ratings of community members, non-physician health workers and doctors in 15 research projects at initial and subsequent assessment. [file 13012_2015_331_MOESM1_ESM.png]
