# Supplementary material for: Behaviour change strategies for reducing blood pressure-related disease burden: findings from a global implementation research programme
Source: Implement Sci. 2015 Nov 9;10:158. doi: 10.1186/s13012-015-0331-0 (PMC4638103; doi:10.1186/s13012-015-0331-0)
Supplement: Additional file 2: — UGA 1 Assessment. Utilizing HIV/AIDS Infrastructure as a Gateway to Chronic Care of Hypertension in Africa - Uganda (UGA 1). [file 13012_2015_331_MOESM2_ESM.pdf]

Utilizing HIV/AIDS Infrastructure as a Gateway to Chronic Care of Hypertension in Africa (UGA 1)

Target behaviour change: Hypertension control

Behaviour change target groups: HIV positive individuals & their families

Country/countries: Uganda

Barriers/enablers to behaviour change

A baseline survey at Mildmay Uganda showed a hypertension prevalence of 27.9% among HIV+ individuals. This is comparable to the anecdotal national level estimate for the general population of 30%. It is estimated that 80% of Ugandans who have hypertension are not aware of their condition. There is no national level data for prevalence or awareness of hypertension among HIV+ individuals. The ratings are therefore based on these national estimates for the general population.

	Community	Non-physician healthworkers	Doctors	Notes
Capability – physical/psychological	Low	Medium	High	1
Motivation – reflective/automatic	Low	Medium	Medium	2
Opportunity – physical/social	Medium	Medium	Medium	3

Notes

1 – See note for ZAF 1.

2 – See note for ZAF 1.

3 – Currently service integration as a strategy to enhance efforts of a weak health system is being encouraged at national level in Uganda. A number of other health services like maternal and child health, and sexual and reproductive health have established community and health facility level support mechanisms that can be used to facilitate behaviour change for NCDs like hypertension. Unfortunately many of these systems are implemented through donor funded projects, bringing to question their sustainability. The wide access to mobile phones and radio networks presents opportunities for wider community reach. Village health teams are in place by national mandate to support information and referral but are only facilitated through projects therefore currently have limited activity beyond specific projects. The health care system is still heavily burdened with communicable diseases, and is grappling with the practicalities of task shifting vs task dumping in the context of high attrition rates for a variety of reasons. Basic screening equipment is still lacking in the facilities and is made worse by the lack of access to free medications after a diagnosis has been made.

Intervention classification

Although there have been a few HIV:hypertension prevalence studies in Uganda, we are not aware of any studies around the barriers/enablers to behaviour change for people with HIV:hypertension co-morbidity. National policy frameworks provide general guidance on hypertension as an important NCD requiring attention but encourage integrated approaches to care without giving specific guidance on how to intervene on barriers/enablers for hypertension treatment in HIV+ individuals.

**Intervention**	GACD project	Notes
Restrictions	N/A	
Education	N/A	
Persuasion	N/A	
Incentivisation	N/A	
Coercion	N/A	
Training	N/A	
Enablement	N/A	
Modelling	N/A	
Environmental restructuring	N/A	1
**Policy factors**		
Guidelines	N/A	
Environment/social planning	N/A	2
Communication/marketing	N/A	
Legislation	N/A	3
Service provision	N/A	4
Regulation	N/A	5
Fiscal measures (eg. taxation)	N/A	

Notes

1 - The NCD Survey which was planned in the first year of the Health Sector Strategic and Investments Plan (HSSIP) 2010/11 - 2014/15, which could have provided an opportunity to model, had not been carried out by 2013, due to lack of funds. HSSIP 2010/11 - 2014/15 monitoring and evaluation indicators, did not have smart indicators for NCDs. These conditions are gradually getting more prevalent in Uganda as a cause of death and poor-health, and their risk factors and interventions are becoming increasingly important. It has been difficult to track, since most data collected is too aggregated for analysis to benefit policy and planning.

2 - *MoH, (1999) National Health Policy. MoH, Kampala. MOH, (2010) Health Sector Strategic and Investments Plan 2010/11 - 2014/15. Promoting People's Health to Enhance Socio-economic Development. MOH Kampala*. The current Ugandan National Health Policy notes that Uganda is simultaneously experiencing a marked upsurge in the occurrence of NCDs such as hypertension and chronic heart disease. The plan documents selective attention to key determinants of ill health in Uganda, including unhealthy lifestyles, despite priority given to infectious diseases. These NCDs have been listed to be among contributors to the burden of disease in Uganda. NCDs will be managed using the Uganda Minimum Health Care package, which comprises interventions that address the major causes of the burden of disease andis the cardinal reference in determining the allocation of public funds and other essential inputs; NCDs fall under essential care in this package. In 2010, the Health Sector Strategic and Investments Plan 2010/11 - 2014/15 prioritised prevention, management and control of NCDs including cardiovascular disease and related diseases. In 2006, The Ugandan Ministry of Health (MoH) established the Programme for the Prevention and Control of NCDs. This small unit is one of nine divisions within the Department of Community Health and is responsible for all national NCD-related activities. A separate division, Health Promotion, is actually responsible for promoting healthy lifestyle changes in the population. According to the Government of Uganda MoH Ministerial Policy Statement 2014/2015, the NCD Programme is allocated 0.01% of the total MoH budget, representing 3% of the Departmental budget, or approximately 27,000USD per annum. The Programme budget is currently supplemented by a five-year grant from the World Diabetes Federation (WDF) that expires at the end of 2017 and that brings the total budget to approximately

270,000USD [unpublished data, Government of Uganda Ministry of Health] – funding remains largely unsustainable with no specific focus on HIV+ populations.

3 - *MOH and WHO (2013). Midterm Review Report of the Health Sector Strategic and Investment Plan (HSSIP) 2010/11 - 2014/15 (VOLUME: 1).* The mid-term review report noted that several awareness campaigns including media and community engagements have been held on NCDs. Camps to enable increased access to specialized care for NCDS has been a best practice.

4 - *MOH and WHO (2013). Midterm Review Report of the Health Sector Strategic and Investment Plan (HSSIP) 2010/11 - 2014/15 (VOLUME: 1).* NCDs have not been given adequate priority in the national agenda, therefore advocacy with the Parliamentary fora on NCDs and Road safety has been done to put the NCD in the national agenda. Sensitization of these group has been done. Cardiovascular diseases guidelines and policies are in draft form and await input from the technical working group.

5 - *MOH and WHO (2013). Midterm Review Report of the Health Sector Strategic and Investment Plan (HSSIP) 2010/11 - 2014/15 (VOLUME: 1).* Regional Referral Hospitals were supported to acquire equipment and training to manage NCDs.
