# Supplementary material for: Behaviour change strategies for reducing blood pressure-related disease burden: findings from a global implementation research programme
Source: Implement Sci. 2015 Nov 9;10:158. doi: 10.1186/s13012-015-0331-0 (PMC4638103; doi:10.1186/s13012-015-0331-0)
Supplement: Additional file 3: — ZAF 1 Assessment. Utilizing HIV/AIDS Infrastructure as a Gateway to Chronic Care of Hypertension in Africa - South Africa (ZAF1). [file 13012_2015_331_MOESM3_ESM.pdf]

Utilizing HIV/AIDS Infrastructure as a Gateway to Chronic Care of Hypertension in Africa (ZAF 1)

Target behaviour change: Hypertension control

Behaviour change target groups: HIV+ individuals

Country/countries: South Africa

Barriers/enablers to behaviour change

Since the present study is still in the data collection stage there is no data on hypertension in the HIV+ population at a national level, but two studies revealed hypertension prevalence of 19%-19.5% which appear to be lower than in the general population. Furthermore, no study has reported on awareness, detection and treatment of hypertension in HIV+ patients in South Africa. These ratings are therefore based on the general population.

	Community	Non-physician healthworkers	Doctors	Notes
Capability – physical/psychological	Low	Medium	High	1
Motivation – reflective/automatic	Low	Medium	Medium	2
Opportunity – physical/social	Low	Low	Low	3

Notes

1 – At a national level, studies showed low awareness of hypertension in the population. It is thought this applies to HIV+ patients also though HIV-infected people regularly access HIV care facilities for their treatment. HIV clinics have not typically provided care for chronic co-morbidities in HIV+ patients due partly to the vertical health care system. Although nurses and community health workers (CHWs) have played a crucial role in providing care at a primary health care level, they often lack the skills to comprehensively deal with NCDs such as hypertension and diabetes. Physicians on the other hand are more equipped for dealing with these diseases.

2 - Cultural influences promoting obesity, a high risk factor for diabetes and hypertension, existing in many South African communities could be a major factor constraining the success of promoting behaviour changes in the communities. The CHWs and nurses have proved to be effective in HIV and TB care, thereby they may also contribute to the success of NCD management in HIV+ patients. Several experimental programmes using CHWs to improve the management of NCDs such as diabetes and hypertension in the general population showed significant results. Like many other under-developed and developing countries, the existing health care system in South Africa is established more for responding to communicable infectious diseases with health staff often having other priorities that compete with NCDs, sometimes leading to a lack of motivation for caring for NCDs among health care staff.

3 - Currently the overburdened and under-resourced health care system is unlikely to be fully meeting patients' needs. Physicians and nurses have insufficient time to do health promotion among patients. CHWs however have greater opportunities to interact with the patients, via home visits for example. Additionally, a report from a recent national survey indicates a low proportion (18%) of participants expressing NCDs as their reason for seeking care at health care facilities. This implicates

underutilization of health care system in prevention and control of NCDs. Among others, a lack of awareness of NCDs in the population may prevent or delay them seeking help.

Intervention classification

**Intervention**	GACD project	Notes
Restrictions	N/A	
Education	N/A	
Persuasion	N/A	
Incentivisation	N/A	
Coercion	N/A	
Training	N/A	
Enablement	N/A	
Modelling	N/A	
Environmental restructuring	N/A	
**Policy factors**		
Guidelines	N/A	
Environment/social planning	N/A	
Communication/marketing	N/A	
Legislation	N/A	
Service provision	N/A	
Regulation	N/A	
Fiscal measures (eg. taxation)	N/A	

Notes

Policy factors:

National guidelines for hypertension and diabetes managements have been produced to assist the health workers in diagnosing and controlling these conditions

The South African food-based dietary guidelines have been developed and disseminated to promote healthy eating among South Africans.

Tobacco legislation has been comprehensively implemented in 2001.

South African ground-breaking salt legislation signed by the Minister of Health, Dr Aaron Motsoaledi on the 18 March 2014 will be put into effect in 2016. This regulation requires salt reductions in processed foods to prevent and reduce hypertension in South African populations

References:

Doherty TM1, Coetzee M (2005). Community health workers and professional nurses: defining the roles and understanding the relationships. Public Health Nurs, 22(4):360-5.

Executive Committee of the Hypertension Society of Southern Africa (1995). Guidelines for the management of hypertension at primary care level. S Afr Med J, 85:1321–5.

Gaziano TA, Bertram M, Tollman SM and Hofman KJ (2014). Hypertension education and adherence in South Africa: a cost-effectiveness analysis of community health workers. BMC Public Health, 14:240. http://www.biomedcentral.com/1471-2458/14/240

Hofman KJ and Tollman SM (2013). Population health in South Africa: a view from the salt mines. The Lancet, 1, e66-67. Available at: http://dx.doi.org/10.1016/S2214-109X(13)70019-6

Malhotra R, Puoane T, Hoyo C, Hughes G, Ostbye T (2008): Prevalence and awareness of hypertension in an urban township of South Africa: compelling need for action. Ethnicity & Disease, 18(4):401-402.

Ndou T, van Zyl G, Hlahane S and Goudge J (2013). A rapid assessment of a community health workers pilot programme to improve the management of hypertension and diabetes in Emfuleni sub-district of Gauteng Province, South Africa. Global Health Action, 6: 19228. http://dx.doi.org/10.3402/gha.v6i0.19228

Peltzer K, Phaswana-Mafuya N (2013).Hypertension and associated factors in older adults in South Africa. Cardiovasc J Afr, 24(3):67-71. doi: 10.5830/CVJA-2013-002.

Puoane T, Tsolekile L, Parker W, Sanders D (2008) Chronic noncommunicable diseases. In: P Barron, J Roma-Reardon (eds) South African Health Review 2008. Durban: Health Systems Trust

Shisana O, Labadarios D, Rehle T et al. (2013). South African National Health and Nutrition Examination Survey (SANHANES-1). Cape Town: HSRC Press. Full report, available at: http://www.hsrc.ac.za/en/media-briefs/population-health/results-sanhanes1

Steyn K and Levitt NS (2006). Health services research in South Africa for chronic diseases of lifestyle. In: Steyn K, Fourie J, Temple N, editors. Chronic diseases of lifestyle in South Africa: 1995-2005, Technical report. Cape Town: South African Medical Research Council. URL: http://www.mrc.ac.za/chronic/cdl1995-2005.pdf

Steyn K, Bradshaw D, Norman R, Laubscher R (2008): Determinants and treatment of hypertension in South Africans: the first demographic and health survey. S Afr Med J, 98(5):376-380

Steyn K, Lombard C, Gwebushe N, Fourie JM, Everett-Murphy K, Zwarenstein M et al. (2013) Implementation of national guidelines, incorporated within structured diabetes and hypertension records at primary level care in Cape Town, South Africa: a randomised controlled trial. Global Health Action, 25(6), 20796. Doi: 10.3402/gha.v6i0.20796.

Working Group of the National Diabetes Advisory Board (1997). Guidelines for the management of type 2 (non-insulin dependent) diabetes mellitus at the primary care level in South Africa. S Afr Med J, 87, 497–512.
