# Supplementary material for: Behaviour change strategies for reducing blood pressure-related disease burden: findings from a global implementation research programme
Source: Implement Sci. 2015 Nov 9;10:158. doi: 10.1186/s13012-015-0331-0 (PMC4638103; doi:10.1186/s13012-015-0331-0)
Supplement: Additional file 4: — COL/MYS 2 Assessment. Developing an Innovative Strategy for Hypertension Detection, Treatment and Control in Two MiddleIncome Countries (Hypertension Outcomes Prevention and Evaluation: HOPE -4) (COL/MYS 2). [file 13012_2015_331_MOESM4_ESM.pdf]

Developing an Innovative Strategy for Hypertension Detection, Treatment and Control in Two Middle Income Countries (Hypertension Outcomes Prevention and Evaluation: HOPE -4) (COL/MYS 2)

Target behaviour change: Improved hypertension control through improved screening, lifestyle changes and medication use

Behaviour change target groups: Urban and rural community residents with hypertension or at high risk for cardiovascular disease

Country/countries: Colombia, Malaysia

Barriers/enablers to behaviour change

	Community	Non-physician healthworkers	Doctors	Notes
Capability – physical/psychological	Medium	Medium	Medium	1
Motivation – reflective/automatic	Medium	Not sure	Medium	2
Opportunity – physical/social	Low	Not sure	Medium	3

Notes

1 - Limited community knowledge regarding hypertension and cardiovascular disease (CVD) and seeking physician care; for the HOPE-4 study, the NPHW will take on a new role and we will assume their knowledge levels are medium; physicians do have the necessary knowledge but do not always prescribe appropriate medication in all eligible hypertension patients.

2 - Partly due to limited knowledge, community participants have limited motivation to adhere to lifestyle changes and medications; we are unsure of NPHWs motivation, their role in CVD prevention is currently likely limited; physicians do not prescribe appropriate medication in all eligible hypertension patients, physicians sometimes regard medication as a treatment to be utilised only after lifestyle change attempts have failed, thus potentially delaying the introduction of drug therapy.

3 - Community residents have no easy access to BP screening, have problems changing lifestyle because of the community's general unhealthy norms, or have problems adhering to medication without family/friend support; for the HOPE-4 study, NPHWs will take on a new role and we are currently unsure of their physical and social opportunities; physicians have limited support from NPHWs in clinic settings and none in community settings, and are traditionally dependent on multiple anti-hypertensive medications that require dose titration to control hypertension

Intervention classification

**Intervention**	GACD project	Notes
Restrictions	No	
Education	Yes	1
Persuasion	Yes	2
Incentivisation	No	
Coercion	No	
Training	Yes	3

Enablement	Yes	4
Modelling	No	
Environmental restructuring	No	
**Policy factors**		
Guidelines	Yes	5
Environment/social planning	Yes	6
Communication/marketing	Yes	7
Legislation	Partially	8
Service provision	Yes	9
Regulation	Not sure	10
Fiscal measures (eg. taxation)	No	

Notes

1 - Participants and treatment supporters will be educated on the effects of risk factors on CVD.

2 - Participants and treatment supporters will be counselled on the effect of improving risk factors on their personal CVD risk, as well as how to achieve such change. NPHWs undertake a specific module focussing on communication techniques to embed improved lifestyle behaviours.

3 - NPHWs will be trained to perform door-to-door screening and risk measurement for hypertension and CVD, to develop treatment plans, and to refer eligible participants to physicians for medications. NPHWs will be trained using a standardized WHO-approved curriculum.

4 - Participants will be enabled to receive appropriate treatment by NPHWs, by measuring BP at home and receiving referral to a physician if needed. Both participant and treatment supporters will be educated to help understand their disease, potential lifestyle modifications and the importance of medication adherence. Physicians (and NPHWs if legally allowed) will be enabled to provide adequate antihypertensive medication by means of access to CVD single-pill combination therapy, which will also enable patients to better adhere to therapy.

5 - Hypertension/CVD management, NPHW training, and participant/supporter education will be tailored to local management guidelines.

6 - Programme should use existing resources (facilities, NPHWs), and facilitate community involvement and access to recommended care in programme.

7 - Importance of hypertension/CVD risk screening, lifestyle and medications will be repeatedly communicated to participants and treatment supporters at NPHW home visits/calls, and at clinic visits. Locally available healthy lifestyle literature will be provided to some participants. Others can opt in for e-health technology (text message and email) treatment and lifestyle reminders

8 - It will be explored whether draft legislation should be provided for countries to facilitate access to combination CVD pills and expand the professional role of NPHWs (medication prescription/changes).

9 - Provision of door-to-door screening, counselling and clinic referral, and CVD single-pill combination therapy currently not available in communities.

10 - If the programme is effective, the plan is to scale up to the regional/national level. Incorporation of the programme into regional/national regulations, taking in account the legislative framework, will be explored during the study but will not be done until the results are known.
