# Supplementary material for: Behaviour change strategies for reducing blood pressure-related disease burden: findings from a global implementation research programme
Source: Implement Sci. 2015 Nov 9;10:158. doi: 10.1186/s13012-015-0331-0 (PMC4638103; doi:10.1186/s13012-015-0331-0)
Supplement: Additional file 5: — CAN 3 Assessment. DREAM-GLOBAL: Diagnosing hypeRtension - Engaging Action and Management in Getting LOwer Bp in Aboriginal and LMIC - Canada (CAN 3). [file 13012_2015_331_MOESM5_ESM.pdf]

DREAM-GLOBAL: Diagnosing hypeRtension - Engaging Action and Management in Getting LOwer Bp in Aboriginal and LMIC (CAN 3)

Target behaviour change: Improved hypertension control through improved screening, lifestyle changes and medication use

Behaviour change target groups: Community health resource (CHRs); primary care providers working with First Nations.

Country/countries: Canada

Barriers/enablers to behaviour change

	Community	Non-physician healthworkers	Doctors	Notes
Capability – physical/psychological	Medium	High	High	1
Motivation – reflective/automatic	Medium	High	Medium	2
Opportunity – physical/social	High	Medium	Medium	

Notes

1 - Community focus groups and key informant interviews with community members, CHRs, and health care providers, and health leadership using the iRREACH tool. CHRs have widely differing capabilities determined by their level of training and experience. CHRs may also be limited by continually increasing expectations and time commitments caused by adding workload from each new programme. The nurses, nurse practitioners and doctors are well trained and capable of taking on new health care behaviours. They are only limited by the scope of their role in that community and time limitations.

2 - Motivation is high for improving the health of the clients/patients, but may be tempered by limits in physical resources to care for them determined by job description limits, space limits and the ability to take on additional workload.

Intervention classification

**Intervention**	GACD project	Notes
Restrictions	No	
Education	Yes	1
Persuasion	No	
Incentivisation	No	
Coercion	No	
Training	Yes	2
Enablement	Yes	3
Modelling	No	
Environmental restructuring	Partially	4
**Policy factors**		
Guidelines	Yes	5
Environment/social planning	Partially	6
Communication/marketing	Yes	7
Legislation	No	

Service provision	Partially	8
Regulation	No	
Fiscal measures (eg. taxation)	No	

Notes

1 - SMS messages provide information to patients twice weekly. Study personnel provide education to local health care providers about the programme and chronic disease management. Attend clinics 2-3 times a year and help answer questions

2 - The CHRs receive training to measure BP and to use the bluetooth and smartphone technology. Participants receive training from the CHRs in the use of their cell phone to receive SMS messages. The CHR is trained on how to respond to BP measures based on a graded system of responses with accessing the emergency medical system for dangerously high BP as an example.

3 - Participants are enabled through the SMS messages to improve their lifestyles. Also to access their health care providers when their BP is uncontrolled. CHRs are enabled to advocate for their clients when BP is uncontrolled. Education about management of chronic disease based on clinical practice guidelines helps all the members of the health care team to work toward the same uniform goal.

4 - Starting with community leadership, approval for the study ensures that all levels of health care providers and health leadership are aware of the programme and are supportive.

5 – Programme is based on the Canadian Hypertension Education Programme (CHEP) clinical practice guidelines.

6 - Using the information gathered with the iRREACH tool, the programme is adapted to the local health care reality.

7 - Hypertension education tools for CHRs, patients, SMS messages to patients on BP management and lifestyle, training manuals for CHRs and other smartphone-based decision support tool for guideline driven management of BP by health care practitioners. Hypertension screening clinics are now scheduled in communities on a regular basis and this has increased awareness in community of the need for control.

8 - Enabling nurses to play a more involved role in individual BP management among patients. Enabling CHRs to enhance their current scope of practice to include more BP assessments and follow up in the community.
