# Supplementary material for: Behaviour change strategies for reducing blood pressure-related disease burden: findings from a global implementation research programme
Source: Implement Sci. 2015 Nov 9;10:158. doi: 10.1186/s13012-015-0331-0 (PMC4638103; doi:10.1186/s13012-015-0331-0)
Supplement: Additional file 6: — TZA 3 Assessment. DREAM-GLOBAL: Diagnosing hypeRtension - Engaging Action and Management in Getting LOwer Bp in Aboriginal and LMIC - Tanzania (TZA 3). [file 13012_2015_331_MOESM6_ESM.pdf]

DREAM-GLOBAL: Diagnosing hypeRtension - Engaging Action and Management in Getting LOwer Bp in Aboriginal and LMIC (TZA 3)

Target behaviour change: Improved hypertension control through improved screening, lifestyle changes and medication use

Behaviour change target groups: Community health workers (CHWs);), community nurses; rural community villagers with hypertension or at risk for hypertension

Country/countries: Tanzania

Barriers/enablers to behaviour change

Notes from community readiness focus groups using our iRREACH tool provided information from consultations sessions with community members etc.

	Community	Non-physician healthworkers	Doctors	Notes
Capability – physical/psychological	Low	Low	Medium	1
Motivation – reflective/automatic	Medium	Medium	Medium	2
Opportunity – physical/social	Low	Medium	High	3

Notes

1 - Limited community knowledge regarding hypertension prevention and control; CHWs have limited knowledge and not trained to assess patients BP and referral; doctors are limited in availability on site to provide continuity in service; staff turn-over is common.

2 - Community medium to high depending on other support or self-efficacy; CHWs not trained to assess BP and unsure of their role and support available to them; physicians (non-MD clinical officers) may refer to lifestyle recommendations or treatment, especially if access to medication or adherence is an issue.

3 - Community doesn’t have access to BP screening or a health worker, may have difficulty with lifestyle changes due access to programmes, healthy eating knowledge, options. CHWs will receive materials to support intervention and assessments, clinical officers will support nurses in prescribing for patients. Clinical officers/physicians likely to prescribe if treatment is accessible and available for patients. Most work done by nurses with clinical officer support and only a few MDs. Opportunity is there if medication readily available which it often isn't so limits ability to improve BP management and patients cannot afford to purchase medication.

Intervention classification

**Intervention**	GACD project	Notes
Restrictions	No	
Education	Yes	1
Persuasion	Partially	2
Incentivisation	Partially	3
Coercion	No	
Training	Yes	4

Enablement	Yes	5
Modelling	No	
Environmental restructuring	Partially	6
**Policy factors**		
Guidelines	Yes	7
Environment/social planning	Yes	8
Communication/marketing	Yes	9
Legislation	Partially	10
Service provision	Partially	11
Regulation	Partially	12
Fiscal measures (eg. taxation)	No	

Notes

1 - Patients will receive SMS messages with lifestyle messages; health care team will receive clinical practice guidelines education on BP management.

2 - Patients will receive SMS messages specific to them (personalized); reminders to see their provider or take their medication.

3 - Patients will receive hypertension treatment at no cost as a result of participation, and this will be transitioned to a partially subsidized programme with patients making a co-payment after month 12 in the study.

4 - Community health workers/community nurses will be trained to assess BP and adhere to follow up assessments and refer to home care nurse/physician if needed.

5 - Patients will receive hypertension treatment as a result of participation, at no cost for 12 months then partially subsidized after that through an e-voucher programme.

6 - Helping the CHWs and nurses to become more involved in patient care and follow up; more assessments, and prompts from SMS messages; nurses will have a decision support tool on their smartphone that is guideline-based and captures historical medication changes and BPs; use of the e-voucher (SMS voucher) for improved drug access

7 - Hypertension management will be from Tanzania HTN 2013 practice guidelines for screening and assessment, treatment to target.

8 - Identify and use existing resources to deliver programme; CHWs, community health centres.

9 - Hypertension education tools for CHWs, patients, SMS messages to patients on BP management and lifestyle, training manuals for CHWs and other smartphone-based decision support tool for guideline driven management of BP by clinical officers and nurses.

10 - Enabling CHWs to enhance their current scope of practice to include more BP assessments and follow up in the community; making drugs more accessible and affordable at dispensaries in Tanzania for patients.

11 - Enabling nurses to play a more involved role in individual BP management among patients. Enabling CHWs to enhance their current scope of practice to include more BP assessments and follow up in the community; making drugs more accessible and affordable at dispensaries and private pharmacies (ADDOS) in Tanzania for patients.

12 - Enabling CHWs to enhance their current scope of practice to include more BP assessments and follow up in the community.
