# Supplementary material for: Behaviour change strategies for reducing blood pressure-related disease burden: findings from a global implementation research programme
Source: Implement Sci. 2015 Nov 9;10:158. doi: 10.1186/s13012-015-0331-0 (PMC4638103; doi:10.1186/s13012-015-0331-0)
Supplement: Additional file 7: — CHN 4 Assessment. A School-Based Education Programme to Reduce Salt Intake in Children and their Families (CHN 4). [file 13012_2015_331_MOESM7_ESM.pdf]

A School-Based Education Programme to Reduce Salt Intake in Children and their Families (CHN 4)

Target behaviour change: Lowered salt intake in children and their families

Behaviour change target groups: Children and families living in Changzhi, Shanxi province, China

Country/countries: China

Barriers/enablers to behaviour change

	Community	Teachers	Doctors	Notes
Capability – physical/psychological	Medium	High	N/A	1
Motivation – reflective/automatic	Medium	High	N/A	2
Opportunity – physical/social	Medium	Medium	N/A	3

Notes

1 - The traditional dietary habits of people in this area of China include very high-salt intake. The general public's knowledge on the harmful effects of salt is poor. There are also misunderstandings, e.g. a reduction in salt intake reduces their physical strength, and some people even believe that salt reduction may cause grey hair. Teachers have similar levels of knowledge about salt as the general community.

2 - When salt intake is initially reduced, there is a sense that the food is bland. Especially in the elderly who have a life time of the same dietary habits, the sudden change in the taste of the food can be difficult to accept. Preventative care visits are less common in China compared to acute visits and the average time of each visit is very short, possibly too short to discuss long-term behaviour changes and health risks. Teachers are highly motivated to engage in health promoting activities.

3 - There are limited resources in this area for preventative health care. In addition, in the rural areas general living standards and income can be low and constrained. There are directives by the education department that require teachers to engage in health related activities.

Intervention classification

**Intervention**	GACD project	Notes
Restrictions	Yes	1
Education	Yes	2
Persuasion	Yes	3
Incentivisation	Yes	4
Coercion	No	
Training	Yes	5
Enablement	Yes	6
Modelling	Yes	7
Environmental restructuring	Yes	8
**Policy factors**		
Guidelines	Yes	9
Environment/social planning	Yes	10
Communication/marketing	Yes	11

Legislation	No	
Service provision	Partially	12
Regulation	No	
Fiscal measures (eg. taxation)	No	

Notes

1 - The intervention includes reducing the amount of salt used during cooking and restricting intake of certain salted foods such as pickles, salted peanuts and other preserved foods, reducing the frequency of eating in restaurants and asking the chefs to reduce salt in their meals when eating out.

2 - The intervention's primary component is an education programme primarily aimed at children, and children were asked to deliver the salt reduction message to adult family members.

3 - The intervention provided materials such as colourful posters and stickers warning against the harms of high salt intake, depicting healthy eating habits, and the methods of reducing salt consumption.

4 - The intervention included prizes for school games and activities involving low-salt topics and certificates for the completion of the programme.

5 - The intervention included skills training to measure salt in cooking (i.e. one bottlecap full is the recommended daily amount of salt intake) and checking labels.

6 - The intervention strongly encouraged children to pass information on to the parents, and to convince them to adopt a reduced-salt diet.

7 - A mini cartoon movie about the salt reduction story of a child's family was used as a model for salt reduction.

8 - The intervention provided posters and stickers that families were encouraged to put in the kitchen or on the fridge so that they would be reminded to eat less salt. Additionally, the project provided a special salt jar and salt spoon to help remind families to eat less salt as well as control the salt used in cooking.

9 – This project used the Chinese government recommended salt intake guidelines (6g/day). The outcome from this project could provide evidence for incorporating salt reduction into CVD prevention guidelines.

10 – We plan to incorporate the education programme into the national school curriculum.

11 – This programme is planned to become part of a larger nationwide salt reduction education programme.

12 - The communication and marketing materials are in the form of a formal classroom curriculum and a low-salt intake book for children.
