# Supplementary material for: Behaviour change strategies for reducing blood pressure-related disease burden: findings from a global implementation research programme
Source: Implement Sci. 2015 Nov 9;10:158. doi: 10.1186/s13012-015-0331-0 (PMC4638103; doi:10.1186/s13012-015-0331-0)
Supplement: Additional file 8: — ZAF 5 Assessment. Treating Hypertension in Rural South Africa: Strengthening Community- Based Outreach Services for Integrated Chronic Care (ZAF 5). [file 13012_2015_331_MOESM8_ESM.pdf]

Treating Hypertension in Rural South Africa: Strengthening Community-Based Outreach Services for Integrated Chronic Care (ZAF 5)

Target behaviour change: Changing clinic systems and behaviour of health professionals in the clinic

Behaviour change target groups: Health professionals, lay health workers, health policy makers, and members of the lay community

Country/countries: South Africa

Barriers/enablers to behaviour change

	Community	Non-physician healthworkers	Doctors	Health administrators	Notes
Capability – physical/psychological	Medium	High	Low	High	1
Motivation – reflective/automatic	Medium	Low	Low	High	2
Opportunity – physical/social	Medium	High	Low	High	3

Notes

1 - Doctors are not very often in the clinics and do not affect how they are run. Observations and interviews in the clinics have noted a wide range in quality of care and motivation of staff. One community focus group reported lack of respect from staff, long waits and drug outages as disincentives. Talks at provincial level indicate very high motivation and support for change.

2 - Involvement of both community members and lay health workers is largely at implementation level. Lay health workers include those with basic secondary certificate and experience in community health work. Doctors are not very often in the clinics. Most of the planning is done at clinic manager’s level and beyond.

3 - For health administrators, there is political will in addressing the growing levels of chronic diseases. The sub-district is one of the beneficiaries of the newly introduced integrated chronic care model. Local non-governmental organisations are already supporting clinics in chronic care, and together with clinic committees are willing to support the intervention

Intervention classification

**Intervention**	GACD project	Notes
Restrictions	No	
Education	Yes	1
Persuasion	Partially	
Incentivisation	No	
Coercion	No	
Training	Yes	2
Enablement	Yes	3
Modelling	No	
Environmental restructuring	Yes	4
**Policy factors**		
Guidelines	No	5

Environment/social planning	No	
Communication/marketing	No	
Legislation	No	
Service provision	Yes	6
Regulation	No	
Fiscal measures (eg. taxation)	No	

Notes

1 - Training for nurses and lay health workers, and health education for patients.

2 - Nurses in-service training and training of lay health workers.

3 - Extra support to enable nurses to function better i.e in service trainings, mobilizing clinic equipment and engaging an implementation manager to support the lay health workers. Also we have provided working BP cuffs since there were none that were useable, and have arranged repair of BP machines.

4 - Altering the patient pathway in the clinic and introducing some activities that were initially not in the clinics i.e. reminding patients of their appointments.

5 - Already in existence but not followed.

6 - Provision of extra resources in clinics.
