# Supplementary material for: Behaviour change strategies for reducing blood pressure-related disease burden: findings from a global implementation research programme
Source: Implement Sci. 2015 Nov 9;10:158. doi: 10.1186/s13012-015-0331-0 (PMC4638103; doi:10.1186/s13012-015-0331-0)
Supplement: Additional file 9: — IND 6 Assessment. Improving the Control of Hypertension in Rural India: Overcoming the Barriers to Diagnosis and Effective Treatment (IND 6). [file 13012_2015_331_MOESM9_ESM.pdf]

Improving the Control of Hypertension in Rural India: Overcoming the Barriers to Diagnosis and Effective Treatment (IND 6)

Target behaviour change: Improved control of BP among rural-dwelling people with hypertension

Behaviour change target groups: Villagers with hypertension, doctors, pharmacists, non-physician health-care workers

Country/countries: India

Barriers/enablers to behaviour change

The first part of our project is to identify the barriers. Some of these have been identified in one or two of the three settings. After the baseline survey of participants and in-depth interviews with providers, we will be more aware of what these factors are. Below we indicate what we suspect may be the barriers.

	Community	Non-physician healthworkers	Doctors	RMPs	Notes
Capability – physical/psychological	Low	Low	Medium	Low	1
Motivation – reflective/automatic	Low	High	Low	Low	2
Opportunity – physical/social	Low	Low	Low	Low	3

Notes

1 - Currently there is low awareness of hypertension in the community in two of the settings (unknown in the third). There is low literacy in all three settings. NPHWs have received minimal training in non-communicable diseases and management. Rural medical practioners (RMPs) have one year of training and their level of knowledge is unclear. Doctors may not always use the appropriate target BP levels.

2 - Doctors have large numbers of patients to see, and while motivated, may not have the capacity. It is unknown what the community demand is. NPHWs are very keen to learn new skills but it is unclear what opportunities exist. It is unclear whether RMPs are motivated or not.

3 - We are unclear how well the health centres are able to assess hypertension (number of blood pressure machines, callibration, training of staff etc.) There is anecdotal information that the government health care providers do not have the equipment or expertise (we aim to find this out). It is unclear what opportunities NPHWs have.

Intervention classification

**Intervention**	GACD project	RAPCAPS*	Kerala study+	Notes
Restrictions	No	No	No	
Education	Yes	Yes	No	1
Persuasion	Yes	No	No	2
Incentivisation	Yes	No	No	3
Coercion	No	No	No	

Training	Yes	Yes	No	4
Enablement	Yes	No	No	5
Modelling	Yes	No	No	6
Environmental restructuring	Yes	Yes	No	7
**Policy factors**				
Guidelines	Yes	Yes	No	8
Environment/social planning	Yes	No	No	9
Communication/marketing	Yes	Yes	No	10
Legislation	No	No	No	
Service provision	Yes	Partially	No	11
Regulation	No	No	No	
Fiscal measures (eg. taxation)	No	No	No	

* RAPCAPS study-Joshi et al JACC 2012

+ Kerala, Thankappan, IJMR 131: 53-63, 2010 gives baseline knowledge but no intervention

Notes

1 - We will use peer-support to educate and monitor patients. There will be an education component for patients, physicians, pharmacists and NPHWs (and potentially next of kin).

2 - Group-based monitoring and education activities have a persuasive component.

3 - We will provide NPHWs with remuneration for following up patients.

4 - Specific skills imparted to participants will be related to accurately taking a BP measurement, use of advice on lifestyles, and medication use. There will also be some health system and workforce strengthening.

5 - Provide opportunities for patients to monitor their BP.

6 - There will be some modelling via the peer-support. People who do well in the fortnightly meetings will be used as exemplars of good outcomes.

7 - Use of a register to prompt NPHWs to contact patients for follow-up.

8 – Participant and NPHWs activities will be targeted towards WHO guidelines for treatment.

9 - We will implement a fortnightly meeting for patients to attend and have their BP assessed.

10 - We will promote the intervention through village leaders.

11 - As per environment/social planning above (see note #9).
