# Supplementary material for: Behaviour change strategies for reducing blood pressure-related disease burden: findings from a global implementation research programme
Source: Implement Sci. 2015 Nov 9;10:158. doi: 10.1186/s13012-015-0331-0 (PMC4638103; doi:10.1186/s13012-015-0331-0)
Supplement: Additional file 10: — IND 7 Assessment. A Smartphone-Based Clinical Decision Support System for Primary Health care Workers in Rural India (IND7). [file 13012_2015_331_MOESM10_ESM.pdf]

A Smartphone-Based Clinical Decision Support System for Primary Health care Workers in Rural India (IND 7)

Target behaviour change: Improved BP control amongst people at high cardiovascular disease risk

Behaviour change target groups: Rural villagers at high risk of CVD, accredited social health activists, doctors

Country/countries: India

Barriers/enablers to behaviour change

This work is informed by previous cross sectional surveys in rural Andhra Pradesh and the findings of the pilot study that we have recently completed.

	Community	Non-physician healthworkers	Doctors	Notes
Capability – physical/psychological	Low	Low	High	1
Motivation – reflective/automatic	Medium	High	Medium	2
Opportunity – physical/social	Low	Low	Low	3

Notes

1 – Currently low awareness of cardiovascular disease (CVD) risk in the community. Low literacy and numeracy in many parts of rural India. Accredited social health activists (ASHAs) have received minimal training in chronic disease prevention and management and have an average of Grade 8 literacy. Doctors are appropriately trained in CVD risk factor management.

2 - Community demand for access to high quality health care is high. ASHAs are strongly encouraged to engage in additional private practice and are very interested in acquiring new skills and employment opportunities to work in chronic disease related areas. Doctors generally express high levels of interest in providing better quality care but may be limited by competing demands on their time.

3 - Currently very limited access to government health care providers and a large proportion of care is provided via private sector practitioners with varying levels of qualifications. ASHAs have few opportunities beyond their current roles in maternal and child health. Primary health care centres have extremely limited capacity to meet patient needs. This is compounded by resource constraints and challenges in efficient use of scarce resources.

Intervention classification

**Intervention**	GACD project	RAPCAPS*	Notes
Restrictions	No	No	
Education	Yes	Yes	1
Persuasion	Yes	No	2
Incentivisation	Yes	No	3
Coercion	No	No	
Training	Yes	Yes	4

Enablement	Yes	No	5
Modelling	No	No	
Environmental restructuring	Yes	Yes	6
**Policy factors**			
Guidelines	Yes	Yes	7
Environment/social planning	No	No	
Communication/marketing	Partially	Yes	8
Legislation	No	No	
Service provision	Partially	Partially	9
Regulation	No	No	
Fiscal measures (eg. taxation)	No	No	

* RAPCAPS study-Joshi et al JACC 2012

Notes

1 - The intervention features separate workforce training programmes for ASHAs and doctors. The decision support application also has embedded educational resources that can be used during the health assessment. In RAPCAPS health workers were provided with education and support to conduct CVD risk assessments.

2 - The multimedia resources have a small persuasive component.

3 - We are considering performance-based remuneration to both ASHAs and doctors to increase uptake of the intervention.

4 - Specific skills in taking an accurate clinical history, use of the tablet device, BP and other CVD risk factor measurement and provision of lifestyle advice. RAPCAPS used a simple paper-based algorithm.

5 - Reducing navigation barriers to getting to the health centre is a key intervention feature.

6 - Use of the tablet and the decision support prompts doctors to consider guideline-based recommendations in an attempt to restructure and systematise workflow.

7 - The decision support algorithm is based on Indian and WHO guidelines.

8 - Some minimal promotion of the study intervention will be done in the intervention villages. RAPCAPS had a community-based health promotion component on salt reduction.

9 - No major changes to current service provision are planned, however some existing aspects of the system will be enhanced (eg. role of ASHAs, electronic referrals).
