# Supplementary material for: Behaviour change strategies for reducing blood pressure-related disease burden: findings from a global implementation research programme
Source: Implement Sci. 2015 Nov 9;10:158. doi: 10.1186/s13012-015-0331-0 (PMC4638103; doi:10.1186/s13012-015-0331-0)
Supplement: Additional file 11: — IND 8 Assessment. Randomised Controlled Trial of Early Use of a Simplified Treatment Regimen Incorporating a Half-Dose, Three-in-One Blood Pressure Lowering Pill vs. Usual Care for Improving Hypertension Control in India (IND8). [file 13012_2015_331_MOESM11_ESM.pdf]

Randomised Controlled Trial of Early Use of a Simplified Treatment Regimen Incorporating a Half-Dose, Three-in-One Blood Pressure Lowering Pill vs. Usual Care for Improving Hypertension Control in India (IND 8)

Target behaviour change: To improve prescriber and patient uptake of BP-lowering medications

Behaviour change target groups: Patients in urban India, doctors

Country/countries: India

Barriers/enablers to behaviour change

	Community	Non-physician healthworkers	Doctors	Notes
Capability – physical/psychological	Medium	N/A	High	1
Motivation – reflective/automatic	Medium	N/A	High	2
Opportunity – physical/social	High	N/A	High	

Notes

1 - Reasonable education, patients attending hospitals clinics with good access to drugs and care. Reasonable income level.

2 - As above for patients. Doctors should be highly motivated to achieve outcomes.

Intervention classification

**Intervention**	GACD project	UMPIRE	Notes
Restrictions	No	No	
Education	No	No	
Persuasion	No	No	
Incentivisation	No	No	
Coercion	No	No	
Training	No	No	
Enablement	Yes	Yes	1
Modelling	No	No	
Environmental restructuring	Yes	No	2
**Policy factors**			
Guidelines	Partially	Partially	3
Environment/social planning	No	No	
Communication/marketing	No	No	
Legislation	No	No	
Service provision	No	No	
Regulation	No	No	
Fiscal measures (eg. taxation)	No	No	

Notes

1 - Making available a simpler, more effective treatment option for BP lowering - three BP-lowering drugs in one. Promoting a simpler up-titration strategy which will minimise requirement for doctor visits for repeated titration/addition of other drugs.

2 - Promoting the availability of a newer drug which has the capability of improving BP control through more rapid reaching of target, convenience of one drug which will promote patient achieving target BP.

3 - We would like to inform guidelines on BP management by contributing to the academic literature around the use of combination medication as first line treatment.
