# Supplementary material for: Behaviour change strategies for reducing blood pressure-related disease burden: findings from a global implementation research programme
Source: Implement Sci. 2015 Nov 9;10:158. doi: 10.1186/s13012-015-0331-0 (PMC4638103; doi:10.1186/s13012-015-0331-0)
Supplement: Additional file 12: — IND 9 Assessment. Developing the Evidence Base for a National Salt Reduction Programme for India (IND 9). [file 13012_2015_331_MOESM12_ESM.pdf]

Developing the Evidence Base for a National Salt Reduction Programme for India (IND 9)

Target behaviour change: Reduction in dietary intake, reduction of salt levels in foods and meals

Behaviour change target groups: Consumers, food industry and service providers

Country/countries: India

Barriers/enablers to behaviour change

	Community	Non-physician healthworkers	Doctors	Food industry and policymakers	Notes
Capability – physical/psychological	Medium	Medium	High	High	1
Motivation – reflective/automatic	Low	Low	Low	Low	2
Opportunity – physical/social	Low	Low	Low	Low	3

Notes

1 - Food Industry and policymakers will have high probability of supporting salt reduction through indirect means. Doctors and community members may have some influence over behaviour change. No empirical work.

2 - Community may have low level motivation due to habit/cultural influences and lack of knowledge about the adverse health impact. No empirical evidence.

3 - Affected by habit and culture and also limited knowledge about the health impact. Controlled by food manufacturers/industry whom without policy push would have little motivation. No empirical evidence.

Intervention classification

**Intervention**	GACD project	China salt substitute	Mongolia workplace intervention	Notes
Restrictions	No	No	No	
Education	Yes	Yes	Partially	1
Persuasion	No	No	No	
Incentivisation	No	No	No	2
Coercion	Not sure	No	No	3
Training	Partially	No	No	4
Enablement	No	No	No	5
Modelling	No	No	No	
Environmental restructuring	Not sure	Yes	Yes	6
**Policy factors**				
Guidelines	Yes	No	No	7
Environment/social planning	No	Not sure	Not sure	
Communication/marketing	Yes	Yes	Yes	8
Legislation	Not sure	No	No	9

Service provision	No	Yes	Yes	
Regulation	Not sure	No	No	10
Fiscal measures (eg. taxation)	Not sure	No	No	11

Notes

1 - Community campaign to build awareness. Provide guidelines on reducing salt intake in food eg WHO and MoH Mongolia work place intervention on salt reduction.

2 - Voluntary or mandated targets for salt levels in foods and/or front of pack labelling.

3 - Potentially taxes on high salt foods.

4 - Training for food industry and food service providers.

5 – Environmental changing – FoodSwitch.

6 - FoodSwitch. Regulations on salt levels.

7 - National guidelines on reducing salt in processed foods/healthy eating guidelines for consumers.

8 - Social marketing campaigns: may cover persuasion/incentivisation.

9 - Potentially targets or labelling may require legislation.

10 - Potentially food industry regulations; voluntary or mandated targets for salt levels in foods.

11 - Potential taxes on high salt foods.
