# Supplementary material for: Behaviour change strategies for reducing blood pressure-related disease burden: findings from a global implementation research programme
Source: Implement Sci. 2015 Nov 9;10:158. doi: 10.1186/s13012-015-0331-0 (PMC4638103; doi:10.1186/s13012-015-0331-0)
Supplement: Additional file 13: — FJI/WSM 10 Assessment. Cost Effectiveness of Salt Reduction Interventions in Pacific Islands (FJI/WSM 10). [file 13012_2015_331_MOESM13_ESM.pdf]

# Cost Effectiveness of Salt Reduction Interventions in Pacific Islands (FJI/WSM 10)

Target behaviour change: Reduce salt use

Behaviour change target groups: Consumers/food industry and service providers

Country/countries: Fiji and Samoa

## Barriers/enablers to behaviour change

| | Community | Non-physician healthworkers | Doctors | Food industry and policymakers | Notes |
|---|---|---|---|---|---|
| Capability – physical/psychological | Medium | High | High | High | 1 |
| Motivation – reflective/automatic | Low | Low | Medium | Medium | 2 |
| Opportunity – physical/social | Low | Medium | Medium | High | 3 |

### Notes

1 - No empirical work to inform rating.

2 - Behaviour is affected by habit and culture and controlled by industry. No empirical work to inform rating.

3 - No empirical work to inform rating.

## Intervention classification

| **Intervention** | GACD project | Mongolia (Ulaanbaatar) workplace intervention on salt reduction | China salt substitute | Notes |
|---|---|---|---|---|
| Restrictions | No | | | |
| Education | Yes | Yes | Yes | 1 |
| Persuasion | No | | | |
| Incentivisation | No | | | |
| Coercion | No | | | |
| Training | Yes | Yes | Yes | 2 |
| Enablement | No | | | |
| Modelling | No | | | |
| Environmental restructuring | Yes | Yes | Yes | 3 |
| **Policy factors** | | | | |
| Guidelines | Yes | Yes | No | 4 |
| Environment/social planning | No | No | No | |
| Communication/marketing | Yes | Yes | Yes | 5 |
| Legislation | No | No | No | |
| Service provision | No | Yes | Yes | 6 |
| Regulation | Yes | No | No | 7 |

| | | | | |
|---|---|---|---|---|
| Fiscal measures (eg. taxation) | No | No | No | 8 |

**Notes**

1 - Consumer campaigns/social marketing strategies

2 - Training for industry and food service providers

3 - Changing salt levels in foods and meals

4 - Policy/strategy to reduce population salt intake to 5g/day

5 - Social marketing campaign

6 - Workplace intervention in Mongolia/Salt substitute in China

7 - Voluntary or mandatory targets for salt levels in food

8 - Potential for taxes on high salt foods but not tried by these countries
