# Supplementary material for: Behaviour change strategies for reducing blood pressure-related disease burden: findings from a global implementation research programme
Source: Implement Sci. 2015 Nov 9;10:158. doi: 10.1186/s13012-015-0331-0 (PMC4638103; doi:10.1186/s13012-015-0331-0)
Supplement: Additional file 14: — PER 11 Assessment. Launching a Salt Substitute to Reduce Blood Pressure at the Population Level (PER 11). [file 13012_2015_331_MOESM14_ESM.pdf]

Launching a Salt Substitute to Reduce Blood Pressure at the Population Level (PER 11)

Target behaviour change: Replacing common salt for a potassium-enriched salt (substitute); incorporating and consuming the salt substitute in the usual diet

Behaviour change target groups: All families accepting to participate in the study, adults aged 18 years and over (mainly women responsible of cooking (housewives)), restaurants and community kitchens

Country/countries: Peru (Tumbes)

Barriers/enablers to behaviour change

	Women responsible for cooking (housewives)	Adults aged 18 years and over	Restaurants and community kitchen	Commercial traders, shopkeepers etc.	Notes
Capability – physical/psychological	Medium	Medium	Medium	Not sure	1
Motivation – reflective/automatic	Medium	Medium	Medium	Not sure	2
Opportunity – physical/social	Low	Low	Low	Low	3

Notes

1 - **Barriers:**

- Resistance to replacement: salt substitute can affect taste of food.
- Potential adverse events of potassium as explained in the informed consent.
- Low levels of knowledge about causes of high blood pressure, especially regarding sodium.

Enablers:

- Potential benefit of salt substitute on health.

2 - **Barriers:**

- Apprehensiveness that it may reduce clients and sales (restaurants and community kitchens).
- Potential intra-familial problems due to impact of salt substitute on food taste.

Enablers:

- Confidence gained by the Center for Global Health (research center of UPCH at Tumbes) that has successfully performed previous interventions.

3 - **Barriers:**

- Lack of availability of the salt substitute in the Peruvian market in Tumbes.
- Lack of access due to high cost of the salt substitute.

Intervention classification

Referring to a marketing campaign to incorporate and guarantee salt substitute consumption in diet of families in the 6 villages of Tumbes. Currently, there is no other similar intervention in Peru. However, several decades ago, there was a large intervention to introduce iodized salt in the diet.

**Intervention**	GACD project	Notes
Restrictions	Partially	1
Education	Yes	2
Persuasion	Yes	3
Incentivisation	Yes	4
Coercion	No	
Training	Yes	5
Enablement	Yes	6
Modelling	Yes	7
Environmental restructuring	Yes	8
**Policy factors**		
Guidelines	No	
Environment/social planning	No	
Communication/marketing	Yes	
Legislation	No	
Service provision	No	
Regulation	No	
Fiscal measures (eg. taxation)	No	

Notes

1 - Salt exchange:

- Exchange all common salt in the household and replace with a salt substitute at the start of the intervention

2 - Marketing campaign:

- Education to guarantee appropriate use of salt in cooking (amounts and standardization), and to promote healthy food preparation spaces (hygiene and adequate use of water, as well as recycling). For this we plan to use the WHO Healthy Municipalities and Communities (HMC) strategy.
- Education about the impact of salt on health: risk and benefits of reducing and replacing salt.

3 - Marketing campaign:

- Creation of a fictional character (‘Mi Amiga Liz’ or ‘My Friend Liz’) who is based on common people living in the villages. She is an expert in cooking and health, guiding participants to introduce the salt substitute in their lives.
- Food fairs in the villages to taste local dishes cooked with our salt substitute; breaking resistance to salt exchange.
- Free availability of salt substitute.

4 - Salt substitute is free and is delivered at household (or close to household); jar (container) and standardized spoons to measure daily salt substitute use; sets for improving kitchen organization.

5 - The training is mainly for personnel of the project or people involved in the intervention:

- Training of health promoters (called ‘Las Amigas de Liz’ or ‘Liz's Friends’) that will work as campaign spokespersons in each village doing face to face communication.
- Training of salt substitute distributors in each village.

6 - Transportation of all ingredients needed from Lima, the capital of Peru, to Tumbes, in the north of Peru. In this way, we guarantee the availability of the product. Manufacturing and packaging process of salt subtitute in Tumbes.

7 - Creation and use of short radio programmes and spots where Liz (main character of the campaign) teaches how to use the salt substitute and suggests how to improve kitchen space with simple tips. Health promoters (called ‘Las Amigas de Liz’ or ‘Liz's Friends’) promoting the use of the salt substitute (disseminating key messages of Liz using flyers and recipes).

8 - Establishing a small factory to prepare the salt substitute (mechanical combination and packaging) for delivery.
