# Supplementary material for: Behaviour change strategies for reducing blood pressure-related disease burden: findings from a global implementation research programme
Source: Implement Sci. 2015 Nov 9;10:158. doi: 10.1186/s13012-015-0331-0 (PMC4638103; doi:10.1186/s13012-015-0331-0)
Supplement: Additional file 15: — GHA 12 Assessment. Task Shifting and Blood Pressure Control in Ghana: A Cluster- Randomized Trial (GHA 12). [file 13012_2015_331_MOESM15_ESM.pdf]

Task Shifting and Blood Pressure Control in Ghana: A Cluster-Randomized Trial (GHA 12)

Target behaviour change: Improved blood pressure control

Behaviour change target groups: Patients with uncontrolled hypertension who receive care in community-based primary care practices

Country/countries: Ghana (Ashanti Region)

Barriers/enablers to behaviour change

No empirical study done yet. Data based on knowledge obtained from nurses during training of implementation of the intervention.

	Community	Non-physician healthworkers	Doctors	Notes
Capability – physical/psychological	Low	Medium	High	
Motivation – reflective/automatic	Low	Medium	Medium	
Opportunity – physical/social	Low	Medium	Medium	

Intervention classification

**Intervention**	GACD project	Notes
Restrictions	No	
Education	Yes	1
Persuasion	Yes	2
Incentivisation	Yes	
Coercion	No	
Training	Yes	3
Enablement	Yes	4
Modelling	Yes	
Environmental restructuring	Yes	5
**Policy factors**		
Guidelines	Yes	
Environment/social planning	Yes	
Communication/marketing	Yes	
Legislation	No	
Service provision	Yes	6
Regulation	Yes	
Fiscal measures (eg. taxation)	No	

Notes

1 - Patient education and training of nurses in patient counselling using motivational interviewing.

2 - Patients were counselled by trained nurses using motivational interviewing.

3 - Nurses were trained in lifestyle behaviour counselling - medication adherence, dietary change, physical activity, and salt intake.

4 - Patients were provided health insurance cards for clinic visits and free medications.

5 - Health insurance cards and role modelling with nurses for training of other nurses.

6 - Provision of health insurance cards and free medications.
