# Supplementary material for: Behaviour change strategies for reducing blood pressure-related disease burden: findings from a global implementation research programme
Source: Implement Sci. 2015 Nov 9;10:158. doi: 10.1186/s13012-015-0331-0 (PMC4638103; doi:10.1186/s13012-015-0331-0)
Supplement: Additional file 16: — KEN 13 Assessment. Optimizing Linkage and Retention to Hypertension Care in Rural Kenya (KEN 13). [file 13012_2015_331_MOESM16_ESM.pdf]

Optimizing Linkage and Retention to Hypertension Care in Rural Kenya (KEN 13)

Target behaviour change:	Linking and retaining hypertensive individuals to hypertensive care
Behaviour change target groups:	Adult individuals with elevated BP (SBP > 140 or DBP >90)
Country/countries:	Kenya

Barriers/enablers to behaviour change

	Community	Non-physician healthworkers	Doctors	Notes
Capability – physical/psychological	Low	Medium	Medium	1
Motivation – reflective/automatic	Medium	Medium	High	2
Opportunity – physical/social	Medium	Medium	Low	3

Notes

1 - Qualitative focus group discussions (FGDs) and mabaraza (traditional Kenyan community assembly): ignorance regarding risks; asymptomatic nature; health education.

2 - Qualitative FGDs and mabaraza: fear of health facility tests and drugs; fear of providers; fear of being burdensome to the family.

3 - Qualitative FGDs and mabaraza: poor service by providers; popularity of alternative medicine; conflicting and competing roles; poverty/lack of money (fare, medical fee, drug fees); unavailability of drugs at health facilities; alcoholism; religious beliefs.

Intervention classification

**Intervention**	GACD project	Notes
Restrictions	No	
Education	Yes	1
Persuasion	Yes	2
Incentivisation	Partially	3
Coercion	No	
Training	Yes	4
Enablement	Yes	5
Modelling	No	
Environmental restructuring	Yes	6
**Policy factors**		
Guidelines	Yes	7
Environment/social planning	No	
Communication/marketing	Yes	8
Legislation	No	
Service provision	Yes	9
Regulation	No	
Fiscal measures (eg. taxation)	No	

Notes

1 - Provision of educational materials relating to healthy living and behavioral change; training of community health workers (CHWs) and community health extension workers (CHEWs).

2 - Tailored behavioural communication strategy: couselling participants on the importance of certain choices and the benefits or disadvantages associated with it; encouraging them to make positive choices and reinforcing with examples.

3 - Considering positive reinforcement for CHWs who successfully link patients to care.

4 – Training CHWs and CHEWs by providing them with the necessary skills in motivational interviewing, smartphone technology, and clinical hypertension assessment.

5 - Providing BP machines to CHWs so as to facilitate the evaluation of BP in the community.

6 - Smartphone tool linked to an electronic health record (in one arm of the trial).

7 - Treatment protocols for hypertension in AMPATH clinics and dispensaries; referral protocols for CHWs.

8 - Mabaraza, discussion with chiefs.

9 - CHWs augmented with a tailored behavioral communication strategy, with or without a smartphone-based tool.
