# Supplementary material for: Behaviour change strategies for reducing blood pressure-related disease burden: findings from a global implementation research programme
Source: Implement Sci. 2015 Nov 9;10:158. doi: 10.1186/s13012-015-0331-0 (PMC4638103; doi:10.1186/s13012-015-0331-0)
Supplement: Additional file 17: — ARG 14 Assessment. Comprehensive Approach for Hypertension Prevention and Control in Argentina (ARG 14). [file 13012_2015_331_MOESM17_ESM.pdf]

Comprehensive Approach for Hypertension Prevention and Control in Argentina (ARG 14)

Target behaviour change: Improved BP control among hypertensive subjects. Targeted behaviour changes include: adherence to hypertensive medication, home blood pressure monitoring and lifestyle modification (weight loss, decrease sodium intake, increase physical activity, reduce excessive alcohol intake, increase potassium intake, and consume a healthy diet - DASH diet)

Behaviour change target groups: Hypertensive patients and their spouses or hypertensive family members

Country/countries: Argentina

Barriers/enablers to behaviour change

	Community	Non-physician healthworkers	Doctors	Health system	Notes
Capability – physical/psychological	Low	Medium	High	Medium	1
Motivation – reflective/automatic	Medium	High	Medium	Medium	2
Opportunity – physical/social	High	High	High	High	3

Notes

1 - Low hypertension awareness and knowledge about hypertension among hypertensive subjects. Community health workers (CHWs) have been trained in and incorporated to maternal and child health care. Primary care physicians have received limited training in approaches to hypertension control. Lack of adherence to treatment guidelines. At the health system level there is increasing interest in community-based care.

2 - Among persons with chronic diseases like hypertension, motivation for BP control is poor because of its silent nature. CHWs are very interested in acquiring new skills to work in chronic diseases. Doctors generally express high levels of interest in providing better quality care but may be limited by competing demands on their time.

3 - Currently, patients with chronic diseases do not receive effective chronic care and continuity of care. Moreover, the national government has launched a public programme (REDES), which provides financial incentives to improve the quality of care at the primary care level. Primary health care clinics have limited capacity to provide high quality care, so the proposed change in the care team with the incorporation of the CHWs, and an active follow-up of hypertensive participants constitutes an opportunity for the province to receive these financial incentives. Hypertensive patients are satisfied with a home intervention that includes self-management education.

Intervention classification

**Intervention**	GACD project	Notes
Restrictions	No	
Education	Yes	1
Persuasion	Yes	2
Incentivisation	Yes	3
Coercion	No	

Training	Yes	4
Enablement	Yes	5
Modelling	No	
Environmental restructuring	Yes	6
**Policy factors**		
Guidelines	Yes	7
Environment/social planning	No	
Communication/marketing	No	
Legislation	No	
Service provision	Yes	8
Regulation	No	
Fiscal measures (eg. taxation)	No	

Notes

1 - Patient education is provided by CHWs during home visits.

2 - Written materials designed for hypertensive patients have a small persuasive component.

3 - We are considering performance-based incentives for CHWs to increase the uptake of the intervention.

4 – CHWs specific skills in providing advice on healthy lifestyle, adherence to antihypertensive medication and home blood pressure monitoring. Also, training was provided to primary care physicians for BP control.

5 - Use of SMS messages to participants as reminders and to support behaviour change. Provide automated home BP monitor and BP log to patients. Work with a public programme to provide free additional antihypertensive medications.

6 - Team change. Incorporation of CHWs into the primary care team to provide chronic care and a home-based intervention. Home delivery of antihypertensive medications.

7 - A decision support algorithm was created based on the JNC8 guideline. This document was used for physician education.

8 - CHWs visit each hypertensive family’s home monthly in the first months and then every two months to provide training and support on lifestyle modification, medication adherence, and BP self-monitoring.
