# Supplementary material for: Behaviour change strategies for reducing blood pressure-related disease burden: findings from a global implementation research programme
Source: Implement Sci. 2015 Nov 9;10:158. doi: 10.1186/s13012-015-0331-0 (PMC4638103; doi:10.1186/s13012-015-0331-0)
Supplement: Additional file 18: — NGA 15 Assessment. Tailored Hospital-based Risk Reduction to Impede Vascular Events after Stroke (THRIVES) (NGA 15). [file 13012_2015_331_MOESM18_ESM.pdf]

Tailored Hospital-based Risk Reduction to Impede Vascular Events after Stroke (THRIVES) (NGA 15)

Target behaviour change: Empowering patients who have had a stroke to improve their adherence to medicines and recommended health care visits

Behaviour change target groups: Patients who have had a past history of stroke and their carers

Country/countries: Nigeria

Barriers/enablers to behaviour change

	Community	Non-physician healthworkers	Doctors	Notes
Capability – physical/psychological	Medium	Medium	High	1
Motivation – reflective/automatic	High	High	High	2
Opportunity – physical/social	Low	Low	Low	3

Notes

1 - Basic literacy levels; high acceptance of the importance of medicines in care (not evenly distributed across all sites).

2 - Training opportunities are high; will to be alive post-stroke is strong; strong social capital with carers expected to play an active role in post-stroke care.

3 - Too many patients to see; lack of technology; patients cannot afford the investigations and medicines; lack of availability of medicines; long distances to travel to get health care; alternative care providers (traditional healers).

Intervention classification

**Intervention**	GACD project	H3Africa – SIREN project	GACD Notes	SIREN Notes
Restrictions	No	No		
Education	Yes	Yes	1	2
Persuasion	Yes	Yes	3	4
Incentivisation	No	No		
Coercion	No	No		
Training	No	Yes		5
Enablement	No	No		
Modelling	No	No		
Environmental restructuring	No	No		
**Policy factors**				
Guidelines	Yes	Yes	6	7
Environment/social planning	No	Yes		8
Communication/marketing	Yes	Yes	9	
Legislation	No	No		
Service provision	No	No		
Regulation	No	No		

Fiscal measures (eg. taxation)	No	No		

Notes

1 - Target behaviour change is promoted via exposure of potential subjects to a brief, but explicit, trilingual in-clinic educational video including compelling segments that are further simplified and delivered by seasoned experts. This video runs in loop and incorporates a touch of African finesse, art and lifestyle.

2 - A first of its kind public initiative to evaluate and address perceptions about stroke and genomics by patients, caregivers and local leaders; incorporating and promoting behavioural modification, medical regimen adherence programmes and community awareness of stroke and its risk factors.

3 - Engagement of a strong and sustained communication (person-person and mobile) strategy to potential subjects thus reinforcing the intervention's resolve to increase their self-efficacy to prevent the occurrence of a repeat stroke event.

4 - Involvement of community gatekeepers, advisory board committee, physicians and research team, in a person-person communication thus nurturing potential subject participation.

5 - Capacity building collaborations are fostered to increase research team's capability in adhering to standard operating practices necessary for the realisation of the study's objectives.

6 - There is strict adherence to stipulated guidelines and policies supporting conduct of clinical research as stated by the study protocol, ethics body and funding organisation.

7 - There is strict adherence to stipulated guidelines and policies supporting conduct of clinical research as stated by the study protocol, ethics body and funding organisation.

8 - Involvement of community gatekeepers in planning, scheduling, community participation, implementation and evaluation of health promotion and screening initiatives.

9 - Promotion of adoption of positive lifestyle behaviours by subjects via dissemination of correct and evidenced-based messages using the mHealth platform.
